# the Improvement Effects of Sika Deer Antler Protein in an Alzheimer's Disease Mouse Model via the Microbe–Gut–Brain Axis

**DOI:** 10.1002/fsn3.4656

**Published:** 2024-12-30

**Authors:** Lei Li, Lulu Wang, Weixing Ding, Jianfa Wu, Fei Liu, Jiansong Liu, Jing Zhang, Jing Wang

**Affiliations:** ^1^ College of Traditional Chinese Medicinal Material Jilin Agricultural University Changchun China; ^2^ School of Medicine Changchun Sci‐Tech University Changchun China; ^3^ Jilin Provincial International Joint Research Center for the Development and Utilization of Authentic Medicinal Materials Changchun China; ^4^ Jilin Province Faw General Hospital Changchun China

**Keywords:** Alzheimer's disease, gut flora, metabolomics, microbe–gut–brain axis, sika deer antler protein

## Abstract

Alzheimer's disease (AD) is a neurodegenerative disorder of the central nervous system. The interplay between the intestinal microbiota and metabolites is believed to influence brain function and the pathogenesis of neurodegenerative conditions through the microbe–gut–brain axis. Sika deer antler protein possesses neuroprotective properties; however, the precise mechanism by which it improves AD remains unclear. Sika deer antler protein ameliorated AD in vivo by activating the phosphatidylinositol 3‐kinase (PI3K)/protein kinase B (AKT)/nuclear factor erythroid 2–related factor 2 (Nrf2) signaling pathway. The metabolome of brain and intestinal tissues and the microbiota of intestinal contents were tested and analyzed according to the microbe‐gut‐brain theory. Sika deer antler protein increased beneficial bacterial levels and decreased harmful bacterial levels. Correlation analyses using the gut flora–metabolomics pathway ultimately revealed that sika deer antler protein modulated the brain and intestinal tract bi‐directionally via the tyrosine metabolism pathway, thereby establishing a connection within the microbe–gut–brain axis. Kyoto Encyclopedia of Genes and Genomes (KEGG) analysis of the differential metabolite targets of the DAP4 group showed that the enriched pathways mainly included PI3K/AKT, which was consistent with the findings of the pharmacodynamic mechanisms observed in in vivo experiments. This suggests that antler protein may be involved in microbe‐gut‐brain interactions through tyrosine metabolism and may improve AD by activating the PI3K/AKT/Nrf2 signaling pathway. These findings add to our understanding of the microbe–gut–brain axis facilitated by sika deer antler protein and offer novel insights for further research on sika deer antler protein in alleviating AD.

## Graphical abstract

The thesis used an in vivo model to evaluate the efficacy of the total protein of sika deer antler and its component DAP4 in improving AD, which may be involved in the interaction between the “microbe–gut–brain” axis through the tyrosine metabolism pathway, activate the PI3K/Akt/Nrf2 signaling pathway, and improve the effect of AD. This provides a new idea for the explanation of the effects of total sika deer antler protein and its component DAP4 on AD.

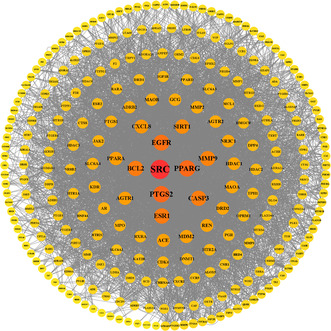

## 1 Introduction

Alzheimer's disease (AD) is a progressive condition that affects the central nervous system and is typically observed in older individuals. The disease is an irreversible chronic dementia disease that accounts for approximately 50%–70% of dementia cases, with a duration of illness usually lasting 5–10 years. Clinical symptoms include memory deficits, visuospatial deficits, and language deficits, which ultimately affect the patient's control over his or her body (Jia et al. 2018; Kolaj, Imindu, and Weaver 2018). Medications sanctioned by the United States Food and Drug Administration for addressing AD primarily consist of acetylcholinesterase inhibitors and N‐methyl‐D‐aspartate receptor antagonists, which are single‐target therapeutic drugs that only alleviate the symptoms and not the disease (Li et al. 2008). As research progresses, the development of AD becomes more intricate and is influenced by numerous factors. This complexity renders it challenging to effectively treat AD using drugs that target a single factor; however, it also unlocks new avenues for research on the development of alternative therapies. Therefore, multitarget drug therapy has become an important direction for drug development. Traditional Chinese medicine (TCM), the main means of Chinese medicinal treatment, possesses the characteristics of multiple pathways and targets and is regarded as a potential strategy for AD treatment.

The microbiome in the intestine and its metabolites are significant factors in AD development. Gut microbes affect gut function and the nervous system through a variety of endocrine, metabolic, and neurological pathways, as well as directly or indirectly by affecting various metabolites. Gut microbes and various metabolites interact through the microbe–gut–brain axis to regulate brain function and the subsequent onset and progression of neurodegenerative diseases (Cryan et al. 2020). Therefore, the study of gut microbes, metabolites, and related pathways in AD is essential and not only contributes to a more comprehensive understanding of the pathophysiology of AD but also helps to identify potential therapeutic targets for AD.

According to the fundamental principles of TCM, the kidneys serve as a repository of essence, permeating the bones and engendering the marrow (Feng et al. 2021). The brain, which is the reservoir of the marrow, establishes a connection between the kidneys and marrow (Feng et al. 2021). AD falls under the classification of dementia and is primarily attributed to insufficiency of kidney essence, thereby leading to a deficiency in the reservoir of marrow, inadequate nourishment of the brain, and an inability to sustain optimal brain function (Shi 2015; Zhu 1959). Consequently, symptoms such as memory loss and delayed cognitive responses manifest. Hence, by employing the therapeutic approach of tonifying the kidney and filling the essence, the objectives of replenishing the kidney essence, enhancing brain nourishment, and promoting brain functionality can be achieved.

The function of the intestinal flora is highly similar to that of the kidney essence in TCM. The kidney is the foundation of the innate essence endowed by parents; it is full of nutrients during pregnancy; and it is perfected during breastfeeding. The colonization of intestinal flora in the human body follows a similar law (Huang et al. 2022; Cheng et al. 2020). The correlation between the two has been discussed by comparing them in terms of source, function, and physiopathological effects, and it is hypothesized that the intestinal flora belongs to the kidney essence (Huang et al. 2022).

The deer antler is the young horn of the unossified and dense hairs of *Cervus nippon* Temminck or *Cervus elaphus* Linnaeus. The antler is warm in nature, sweet and salty in taste, and is attributed to the liver and kidney meridians (National Pharmacopoeia Commission 2020). Deer antlers are rich in polysaccharides such as proteins, amino acids, and minerals. These components are inseparable from deer antler quality characteristics and can be used to determine deer antler quality and to study their medicinal efficacy. The sika deer antler has a variety of pharmacological activities, including nervous system protection, antioxidant activity, and intestinal flora improvement. Approximately 144 proteins were highly expressed in *C. nippon* sika deer antler, whereas only 36 proteins were highly expressed in *C. elaphus* deer antlers, and the protein content was significantly higher than that of *C. elaphus* Linnaeus deer antler. Further gene ontology and KEGG analyses indicated that the differentially expressed proteins play a role in pathways such as the phosphatidylinositol 3‐kinase (PI3K)/protein kinase B (AKT) signaling pathway and oxidative phosphorylation (Yang 2020). A key component of sika deer antler is the antler protein, which has neuroprotective properties. However, the exact mechanism by which sika deer antler protein improves AD remains unclear. Based on the multi‐target and multi‐pathway therapeutic properties of TCM, we chose sika deer antler protein as the focus of this study. We explored the mechanism by which sika deer antler proteins link the intestines with the brain via the intestinal microorganisms. This was based on the theory of tonifying the kidney and replenishing the essence, the hypothesis that intestinal flora belongs to the kidney essence, and the microbe‐gut‐brain theory. We also explored methods to improve AD through bi‐directional regulation, which has important significance for the clinical treatment of AD.

In this study, we screened active fractions of sika deer antler proteins with ameliorative effects on AD using an okadaic acid (OA)‐induced mouse hippocampal cell (HT22) injury model. We investigated the mechanism of action of the active fractions screened in vitro to ameliorate AD using an AlCl_3_/D‐gal‐induced AD mouse injury model. Using the microbe–gut–brain axis theory, we combined 16S rRNA high‐throughput sequencing technology with metabolomics to explore the mechanism by which sika deer antler proteins improve AD.

## 2 Materials and Methods

### 2.1 Materials, Reagents, and Instruments

#### 2.1.1 Experimental Materials

The total protein of sika deer antler (DAP) was separated in‐house using Sephadex G‐100 to obtain different protein fractions: DAP1, DAP2, DAP3, DAP4, and DAP5 (Ruan et al. 2019).

#### 2.1.2 Experimental Reagents

OA was purchased from Shanghai Yuanye Biotechnology Co. Ltd., LY 294002 inhibitor was purchased from GLPBIO, AlCl_3_ and D‐gal were purchased from Shanghai McLean Biochemical Technology Co. Ltd., and hematoxylin and eosin (HE) stain was purchased from Shanghai Yuanye Biotechnology Co. Ltd. Biochemical kits for superoxide dismutase (SOD), glutathione (GSH), and malondialdehyde (MDA) were purchased from Shenyang Wanban Biological Company, and amyloid beta (Aβ)_1–42_, acetylcholinesterase (AChE), acetylcholine (ACh), and 5‐hydroxytryptamine (5‐HT) enzyme‐linked immunosorbent assay (ELISA) kits were purchased from Jiangsu Jingmei Biotechnology Co. The Pv‐6001 and 3,3′‐diaminobenzidine (DAB) kits were purchased from Beijing Zhongsugi Jinqiao Biotechnology Co. Rabbit nuclear factor erythroid 2–related factor 2 (Nrf2), Kelch‐like ECH‐associated protein 1 (keap1), heme oxygenase 1 (HO‐1), NAD(P)H quinone dehydrogenase 1 (NQO1), B‐cell lymphoma 2 (Bcl‐2), Bcl‐2 associated X (Bax), Caspase 3, Caspase 9, cytochrome C (CytoC), PI3K, and AKT antibodies and horseradish peroxidase (HRP)‐labeled goat anti‐rabbit immunoglobulin (Ig)G (H + L) were purchased from Shenyang Wanclass Biological Company. The phosphorylated PI3K (*p‐*PI3K) and phosphorylated AKT (*p‐*AKT) antibodies were purchased from Chengdu Zheneng Biotechnology Co. Ltd., and the Aβ_1‐42_, ionized calcium‐binding adapter molecule 1 (Iba1), and *p‐*Tau antibodies were purchased from Beijing Boao Sen Biotechnology Co. Ltd. The glial fibrillary acidic protein (GFAP) antibody was purchased from Wuhan Doctoral Biological Engineering Co. Ltd., and a GC buffer‐containing Phusion high‐fidelity polymerase chain reaction (PCR) mixture and Phusion high‐fidelity DNA polymerase were purchased from New England Biolabs (USA). The PCR product DNA purification kit was purchased from Tiangen Biochemical Technology Co., and methanol and ammonia were purchased from Thermo Fisher Scientific Ltd.

#### 2.1.3 Laboratory Instruments

The following instruments were used: PL303 electronic balance (Mettler Toledo Technologies Inc.); ST16R high‐speed refrigerated centrifuge (Thermo Fisher Scientific, USA); T10 basic S25 handheld high‐speed homogenizer (IKA, Germany); BioTek epoch full wavelength enzyme labeler (BioTek Instruments Inc., USA); WMT‐100 water maze video analysis system, OFT‐100 open field activity experiment box, RMT‐100 eight‐arm maze (Chengdu Taimeng Technology Co. Ltd.); TB‐718E biological tissue automatic embedding machine (Hubei Taiwei Science and Technology Industry Co. Ltd.); electrophoresis device, Tanon 5200 chemiluminescence imaging analysis system (Shanghai Tianneng Technology Co.); RM2235 paraffin sectioning machine, DM2500 light‐emitting diode ortho optical/fluorescence microscope (Leica Microsystems AG, Germany); Qubit 2.0 type quantum bit fluorescence quantification (Thermo Fisher Scientific, USA); Agilent 5400 bioanalyzer (Agilent Technologies Inc., USA); T100 PCR instrument (Bio‐Rad, USA); sequencer (Illumina, USA); Q Exactive mass spectrometer and Vanquish ultra‐high‐performance liquid chromatography (UHPLC) ultra‐high pressure liquid chromatograph (Thermo Fisher Scientific, USA).

### 2.2 Methods

#### 2.2.1 Amelioration of OA‐Induced HT22 Damage by Sika Deer Antler Total Protein and Protein Fractions

HT22 cells were cultured in 96‐well plates at a density of 1 × 10^5^ cells/well and incubated for 24 h. Cells were co‐incubated with different concentrations of DAP, DAP1, DAP2, DAP3, DAP4, and DAP5 for 24 h. The cells were treated with OA (40 nmol/L) for 24 h to simulate specific conditions. Cells were treated with 0.5 mg/mL 3‐(4,5‐dimethylthiazol‐2‐yl)‐2,5‐diphenyl‐2H‐tetrazolium bromide (MTT) and incubated for 4 h. The solution was then aspirated, and DMSO (150 μL per well) was added to dissolve and produce blue‐purple crystalline A. The absorbance value was read at 490 nm using an enzyme counter, and cell viability was calculated.

#### 2.2.2 Animals and Treatment

Six‐week‐old specific pathogen‐free‐grade male Kunming mice (18–22 g) were acquired from Changchun Yisi Laboratory Animal Technology Co. Ltd. (Quality Certificate No. SCXK (JI) 2020‐0001; Changchun, China). The mice were housed in a standard laboratory environment at the College of Traditional Chinese Medicine, Jilin Agricultural University, in a regulated setting where the temperature and humidity were at 22°C ± 2°C and 55% ± 10%, respectively, with a 12‐h light/dark cycle. The mice were provided with unlimited food and water and allowed to acclimate for 7 days. A total of 60 animals were used in the experiment, with 10 animals in each group after they were acclimated. The mice were then randomly assigned to six groups: Normal, Model, DAP (200 mg/kg), DAP (400 mg/kg), DAP4 (100 mg/kg), and DAP4 (200 mg/kg). The normal group received 0.9% saline solution, and the model group was administered AlCl_3_ (20 mg/kg) by gavage, and D‐gal (200 mg/kg) was injected intraperitoneally for 8 weeks. In the drug administration groups, AlCl_3_ (20 mg/kg) was administered by gavage, D‐gal (200 mg/kg) was injected intraperitoneally, and DAP (200 mg/kg or 400 mg/kg) and DAP4 (100 mg/kg or 200 mg/kg) were administered by gavage for 8 weeks. Behavioral analysis was performed after 8 weeks of administration.

#### 2.2.3 Behavioral Tests

##### 2.2.3.1 Eight‐Arm Maze

After the animals were acclimatized to the experimental environment, they were fasted for 24 h, and only a restricted supply of normal food was available at the end of each training day. Upon completion of the training, four arms were randomly selected, and one food pellet was placed in the food box of each arm. During each set of experiments, the mice were positioned in the central region of the labyrinth to ensure free movement and pellet ingestion until food pellets from all four arms were ingested, after which the test was completed. The experiment was terminated if the mice did not consume the food pellets after 5 min. During the experiment, the mice's action trajectories were tracked using RMT‐100 software in the TM‐Vision Behavioral Experiment System.

##### 2.2.3.2 Morris Water Maze (MWM)

The MWM test assesses the spatial abilities of mice by swimming in a water‐filled pool to locate a hidden platform. The setup included a cylindrical pool, opaque water, a platform, and a behavior analysis system. The WMT‐100 Image Acquisition and Analysis System recorded mouse swimming track data to extract and analyze indicators. The localization navigation experiment required training the mice to find a movable platform in the center of a fixed quadrant. If the mice failed to locate the platform within a set timeframe, they were directed to the right location and given a 30‐s wait before recording the time it took to find the hidden platform. Following the localization navigation experiment, the spatial exploration study eliminated the initial platform and monitored the frequency of mice crossing the platform, along with their duration of stay.

#### 2.2.4 Serum Sample Preparation

After the mice completed all behavioral tests, they were fasted for 12 h, and blood samples were collected in centrifuge tubes by removing the eyeballs of the mice. After the blood was placed in a centrifuge tube for a period of time, it was centrifuged at 13400 g for 10 min at 4°C, and the supernatant was collected; this step was repeated twice. Ultimately, pure serum samples were obtained, then distributed and preserved in a freezer at −80°C for future use.

#### 2.2.5 Preparation of Brain Tissue Homogenate

Mice were dissected after blood sampling, and brain tissues were collected. Parts of the brain tissues were preserved in tissue fixative for paraffin embedding, whereas other parts were wrapped with tinfoil, frozen in liquid nitrogen, and stored at −80°C to prepare brain tissue homogenate.

Brain tissue of a certain size was collected and crushed with a tissue homogenizer on ice at a ratio of brain tissue (g) to saline (mL) of 1:9 with uniform mixing. After the homogenization was completed, centrifugation was performed at 12,000 rpm and 4°C for 15 min, and the supernatant, which was the brain tissue homogenate, was collected and stored at −80°C for spare use.

#### 2.2.6 Histopathology Staining Analysis

Brain tissue was removed from the tissue fixative and embedded in paraffin after gradient ethanol dehydration and xylene hyalinization. After the paraffin blocks were cooled, they were placed on a rotary paraffin slicer and sliced into 4‐μm‐thick sections. Brain sections were stained using a commercially available HE staining kit, and pathological changes in mouse hippocampal neurons were then observed using an orthogonal light/fluorescence microscope.

#### 2.2.7 Determination of Aβ_1–42_ Levels

Serum and brain tissues were treated according to the preparation methods described in Sections 2.2.4 and 2.2.5, respectively, and the ELISA kit procedure was followed to determine the Aβ_1–42_ levels in the samples. The absorbance at 450 nm was measured using an enzyme meter.

#### 2.2.8 Oxidative Stress Indicator Test

Brain tissues were processed according to the preparation method described in Section 2.2.5, and the MDA, total SOD (T‐SOD), and GSH levels in the brain tissues were determined according to the kit procedure. The absorbance values were determined using an enzyme meter.

#### 2.2.9 Neurochemical Analysis

Serum and brain tissues were processed according to Sections 2.2.4 and 2.2.5, respectively. An ELISA kit was used to determine the AChE, ACh, and 5‐HT levels in the serum and brain tissue, and the absorbance values were read at 450 nm using an enzyme meter.

#### 2.2.10 Immunohistochemical Analysis

Brain tissues were removed from the tissue fixative and embedded in paraffin wax after gradient ethanol dehydration and xylene permeabilization, and the embedded tissues were sliced into 4‐μm‐thick sections. The segments were immersed in a 0.01 M sodium citrate solution (pH 6.0) for 30 min to repair the antigen, followed by three washes with pre‐cooled phosphate‐buffered saline (PBS) buffer for 5 min each. The sections were closed for 20 min at room temperature by dropwise addition of normal goat serum, and the excess liquid was shaken off. The sections were incubated with primary antibodies Aβ_1–42_, *p*‐Tau, Iba1, GFAP, Nrf2, and HO‐1, respectively, and then left to incubate overnight at 4°C. The following day, the sections were rinsed thrice with PBS for 5 min. After incubating the sections with the secondary antibody at 37°C for 20 min, they were washed thrice with PBS for 5 min each. Finally, the color was developed using the DAB kit, sealed with neutral gel, and observed under a light microscope.

#### 2.2.11 Western Blot

Mouse brain tissue was lysed with RIPA lysis solution containing a protein phosphatase inhibitor, followed by centrifugation at 12,000 rpm for 15 min at 4°C to extract total protein samples. After extracting the supernatant, the protein concentration was calculated using a bicinchoninic acid assay kit. The samples were subjected to protein quantification using a loading buffer and PBS and were boiled at 100°C for 10 min to completely denature the proteins in the samples.

After protein quantification and boiling, protein samples were separated on a 12% sodium dodecyl‐sulfate polyacrylamide gel electrophoresis separation gel and a 5% concentrated gel. Following separation, the proteins from the gel were electrophoretically transferred onto a polyvinylidene fluoride membrane, which was then incubated with 5% skim milk for 2 h. The membrane was washed thrice with 1X Tris‐buffered saline with 0.1% Tween 20 detergent (TBST) for 12 min each. The membrane was then incubated at 4°C overnight with different primary antibodies, including Nrf2 (1:800), HO‐1 (1:800), NQO1 (1:800), keap1 (1:1200), Bax (1:800), Bcl‐2 (1:500), Caspase 3 (1:800), Caspase 9 (1:800), CytoC (1:1500), AKT (1:800), PI3K (1:1200), *p‐*AKT (1:1200), *p*‐PI3K (1:800), and β‐actin (1:800). The following day, the primary antibody was collected and rinsed thrice using 1X TBST for 12 min each. The membrane was then incubated with HRP‐labeled goat anti‐rabbit IgG (H + L) secondary antibody (1:5000) for 1.5 h at room temperature and rinsed with 1X TBST. The signal was then identified using electrochemiluminescence luminescent solution. Signal intensity was examined using Image‐Pro plus 6.0 software.

#### 2.2.12 DNA Extraction and High‐Throughput 16S rRNA Sequencing

After extracting total DNA from the intestinal contents in strict accordance with the kit instructions, the V3‐V4 region of the extracted DNA samples was amplified using PCR with the 314F (CCTAYGGGGRBGCASCAG) and 806R (GGACTACNNGGGTATCTAAT) primers. After purifying the PCR‐amplified products, a NovaSeq 6000 PE25 platform was used for high‐throughput sequencing. After up‐sequencing, the bipartite data were spliced, quality‐controlled, and chimera‐filtered using the overlap method to obtain high‐quality CleanData, which were analyzed for species, diversity, and significantly different genera after noise reduction using R4.3.1, and de‐duplication using DADA2 software.

#### 2.2.13 Metabolomics

The brain tissue and intestinal tissue samples were thawed slowly at 4°C, then placed in a solution of methanol/acetonitrile/water (2:2:1 ratio). The solution was vortexed, sonicated at low temperature for 30 min, incubated at −20°C for 10 min, and then centrifuged at 14,000 rpm at 4°C for 20 min. The supernatant was collected and dried under a vacuum. For mass spectrometry analysis, 100 μL of aqueous acetonitrile solution (1:1 ratio) was used for resolution, vortexed, mixed, and centrifuged at 15633 g at 4°C for 15 min. The resulting supernatant was injected and subsequently analyzed.

The samples were separated on a Thermo Vanquish UHPLC ultra‐high performance liquid chromatography (UPLC) system (Waters UPLC BEH Amide column, 2.1 × 100 mm, 1.7 μm; Thermo Fisher Scientific), and the primary and secondary spectra were collected and analyzed using a Q Exactive mass spectrometer (Thermo Fisher Science). The separation using UHPLC used a column temperature set at 25°C, a flow rate of 0.3 mL/min, and an injection volume of 2 μL. Moreover, a water +25 mM ammonium acetate +25 mM ammonia A and acetonitrile B solution was used. The gradient elution program was 0–1.5 min, 2% A, 98% B; 1.5–12 min, 2%–98% A, 98%–2% B; 12–14 min, 98% A, 2% B; 14–14.1 min, 98%–2% A, 2%–98% B; and 14.1–17 min, 2% A, 98% B. The parameters for the electrospray ionization source and mass spectrometry were as follows: Nebulizing gas auxiliary heating gas 1 (Gas1), 60 psi; auxiliary heating gas 2 (Gas2), 60 psi; curtain gas (CUR), 30 psi; ion source temperature, 600°C, and spray voltage (ISVF) ± 5500 V (positive and negative modes); primary mass‐to‐charge ratio detection range, 80–1200 Da; resolution, 60,000; scan accumulation time, 100 ms; second stage adopted the segmented acquisition method with a scanning range of 70–1200 Da; second stage resolution, 30,000; scanning accumulation time, 50 ms; and dynamic exclusion time, 4 s. During the analysis, the sample was loaded into an autosampler with a sample well set at 4°C. To prevent the impact of fluctuations on the instrument detection signal, the samples were analyzed in a random sequence without interruption. Quality control samples were added to the sample lineup to assess the stability and accuracy of the experimental results.

#### 2.2.14 Statistical Analysis

All experimental data were analyzed and compared between groups using mean ± standard deviation (mean ± SD). Analysis of variance (ANOVA) software was used for statistical analysis, ImageJ software for quantitative image analysis, and GraphPad Prism software (version 8.0) for graphical data plotting. Statistical significance was indicated by *p* values < 0.01 or 0.05.

For quantitative analysis of metabolites, the sample data were analyzed based on quality control, principal component analysis (PCA), partial least squares‐discriminant analysis (PLS‐DA), and volcano plots in R language. The metabolites that met log fold change (|logFC|) > 0.5, *p* < 0.05, and variable importance of projection (VIP) ≥ 1.0 were selected as the differential metabolites. For the metabolites obtained from the screening, the metabolic pathway information provided by KEGG was analyzed using the Metabo Analyst 5.0 tool to obtain significantly enriched metabolic pathways, with *p* < 0.05 as the threshold for significant enrichment. Significant similarity clustering of metabolite abundance patterns was performed using Short Time‐series Expression Miner (STEM) software (similarity threshold: 0.8, significance threshold: *p* < 0.05), and the metabolites contained in the clustering patterns of interest were selected for analysis. Finally, the metabolites with overlapping differential distributions obtained from the screened differential clusters and STEM analyses were used to calculate the correlation between them using the cor function Spearman algorithm in R4.3.1. A significance of *p* < 0.05 and an absolute value of the correlation coefficient higher than 0.4 were selected as the screening threshold. The retained metabolites with significant correlation with the colony were then subjected to KEGG signaling pathway annotation analysis, and a Sankey diagram of the relationship network of the colony‐differentiated metabolite pathway was constructed.

## 3 Results

### 3.1 Amelioration of OA‐Induced HT22 Cell Damage by Sika Deer Antler Total Protein and Protein Fractions

**FIGURE 1:**
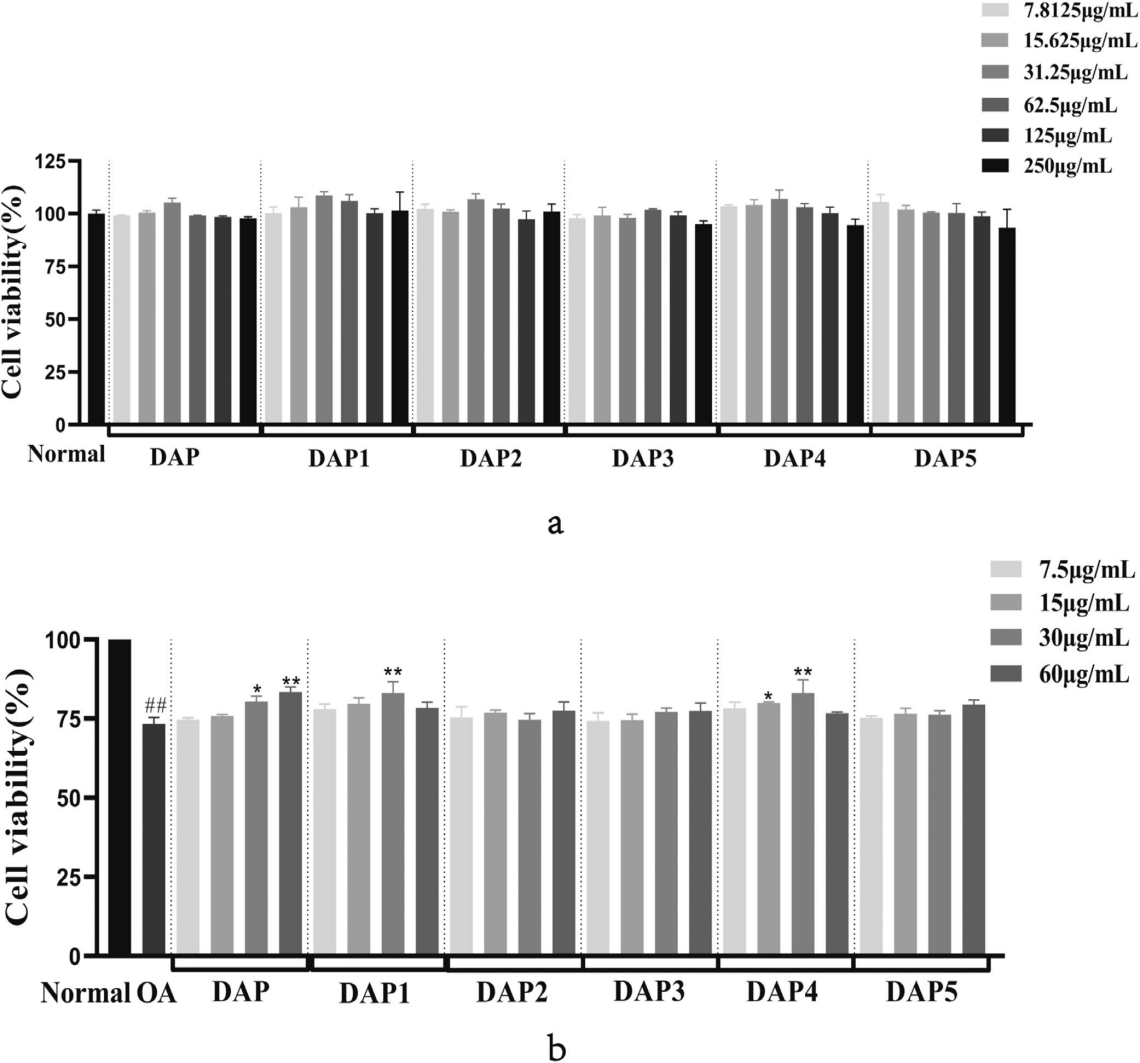
The effects of sika deer antler total protein and protein components on HT22 cells. ^#^ *p* < 0.05, ^##^ *p* < 0.01 compared with that of the normal group; *p < 0.05, **p < 0.01, compared with that of the model group. (a) Effects of sika deer antler total protein and protein components on HT22 cell proliferation. (b) Effects of sika deer antler total protein and protein components on OA‐induced HT22 cell damage.

The MTT assay was used to measure cell viability, and the cell proliferation test results are displayed in Figure 1a. The various protein fractions from sika deer antler did not exhibit any harmful effects on HT22 cells. Figure 1b shows that DAP concentrations between 30 and 60 μg/mL notably enhanced cell viability reduction in OA‐induced HT22 cells compared with that in the OA model group. Moreover, DAP4 significantly increased the OA‐induced decrease in HT22 cell viability in the dose range of 15–30 μg/mL. Based on the screening outcomes, DAP and DAP4 were selected for further testing.

### 3.2 Effects of DAP and DAP4 on Cognitive Impairment in AD Model Mice

**FIGURE 2:**
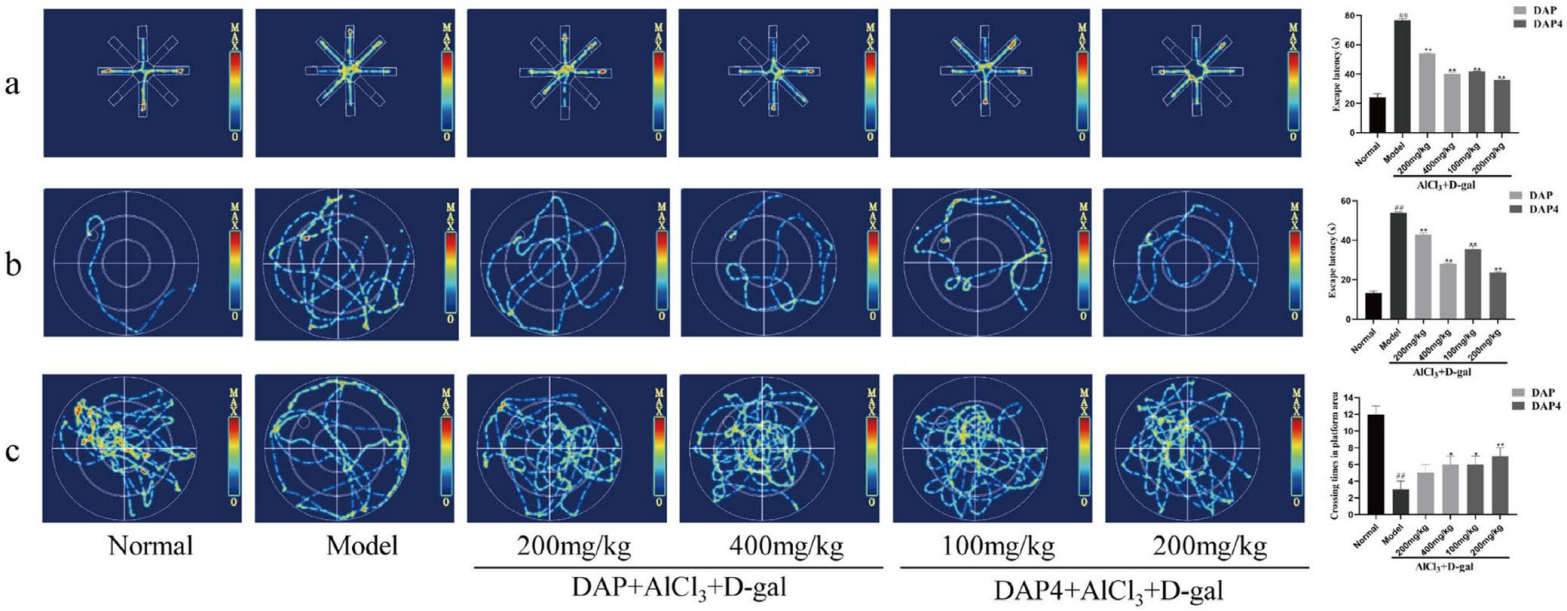
Effects of DAP and DAP4 on the behavior of AlCl_3_/D‐gal‐induced AD mice. ^#^ *p* < 0.05, ^##^ *p* < 0.01 compared with that of the normal group; *p < 0.05, **p < 0.01, compared with that of the model group. (a) Eight‐armed maze action trajectory and escape latency. (b) Navigation test swim trajectories and escape latencies (c) Probe test swim trajectory and number of platform crossings.

The memory capacity of the mice in the eight‐arm maze was assessed by comparing the time taken for the mice to find and consume the four food pellets placed in different arms of the maze. The results are shown in Figure 2a. Mice in the model group showed a significant increase in escape latency compared with those in the control group. Additionally, the administration of DAP and DAP4 significantly reduced escape latency.

The mice were tested for memory and spatial learning using the MWM test to determine whether they had any cognitive impairments. As shown in Figure 2b, in the navigation test experiment, the escape latency to reach the hidden platform was longer in the AlCl_3_/D‐gal model group than in the control group, indicating that spatial memory was significantly impaired in AD mice. DAP and DAP4 administration significantly reduced escape latency in mice. As shown in Figure 2c, in the probe test experiment, the number of times the AlCl_3_/D‐gal model group traversed the platform in the target quadrant was significantly lower in the AlCl_3_/D‐gal model group than in the control group, and DAP and DAP4 administration increased the number of times the mice traversed the platform in the target quadrant. Therefore, the MWM tests revealed that AlCl_3_/D‐gal administration impaired spatial learning and memory and that sika deer antler protein restored cognitive function and improved spatial learning and memory in AD mice.

### 3.3 Effects of DAP and DAP4 on Histopathologic Changes in the Brain of AD Mice

**FIGURE 3:**
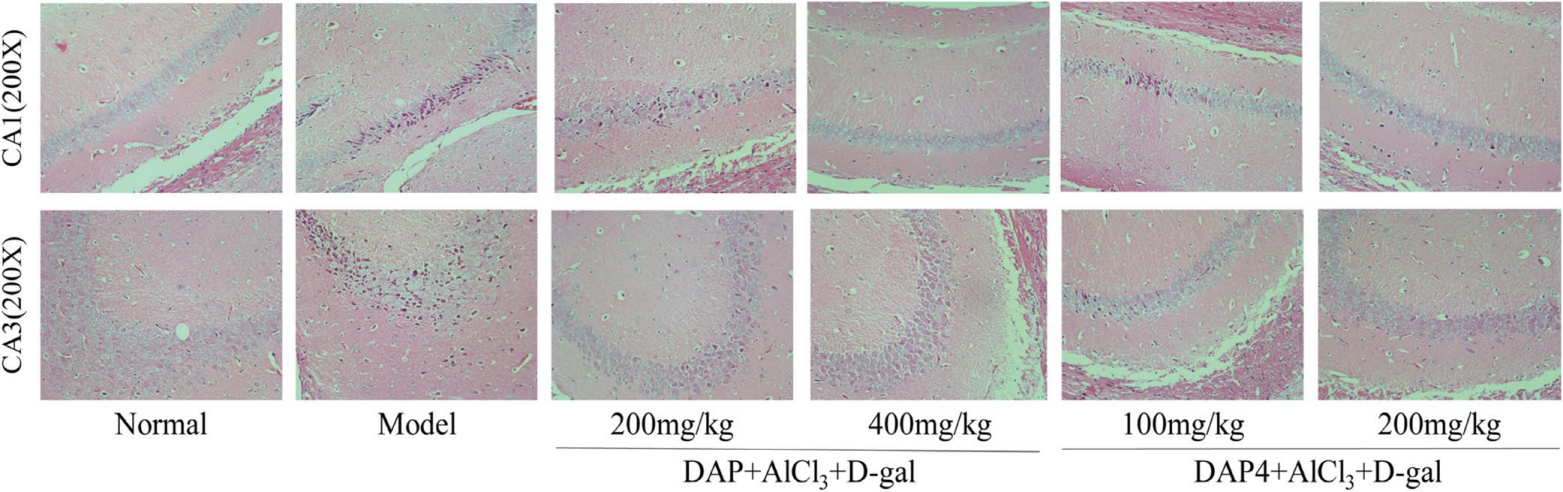
The effects of DAP and DAP4 on the pathological changes of hippocampal tissue in AlCl_3_/D‐gal‐induced AD mice.

Neurons are essential for cognitive functions such as learning and memory. HE staining was used to analyze alterations in neurons in the CA1 and CA3 areas of the hippocampus to study whether DAP and DAP4 impact neuronal morphology. Figure 3 shows that the hippocampal neurons of the control group were well preserved, exhibiting distinct cell structures and nuclei and a dense and orderly arrangement of cells. In comparison with the AlCl_3_/D‐gal model group, DAP and DAP4 improved morphological alterations in the CA1 and CA3 areas of the damaged hippocampus, showing reduced neuronal damage, preserved nuclear membranes in the hippocampal area, and a high quantity of intact and clearly defined neurons closely packed in a circular pattern with minimal space. This suggests that DAP and DAP4 improve AlCl_3_/D‐gal‐induced neuronal damage.

### 3.4 Effects of DAP and DAP4 on Aβ_1–42_ and *p*‐Tau Levels in AD Mice

**FIGURE 4:**
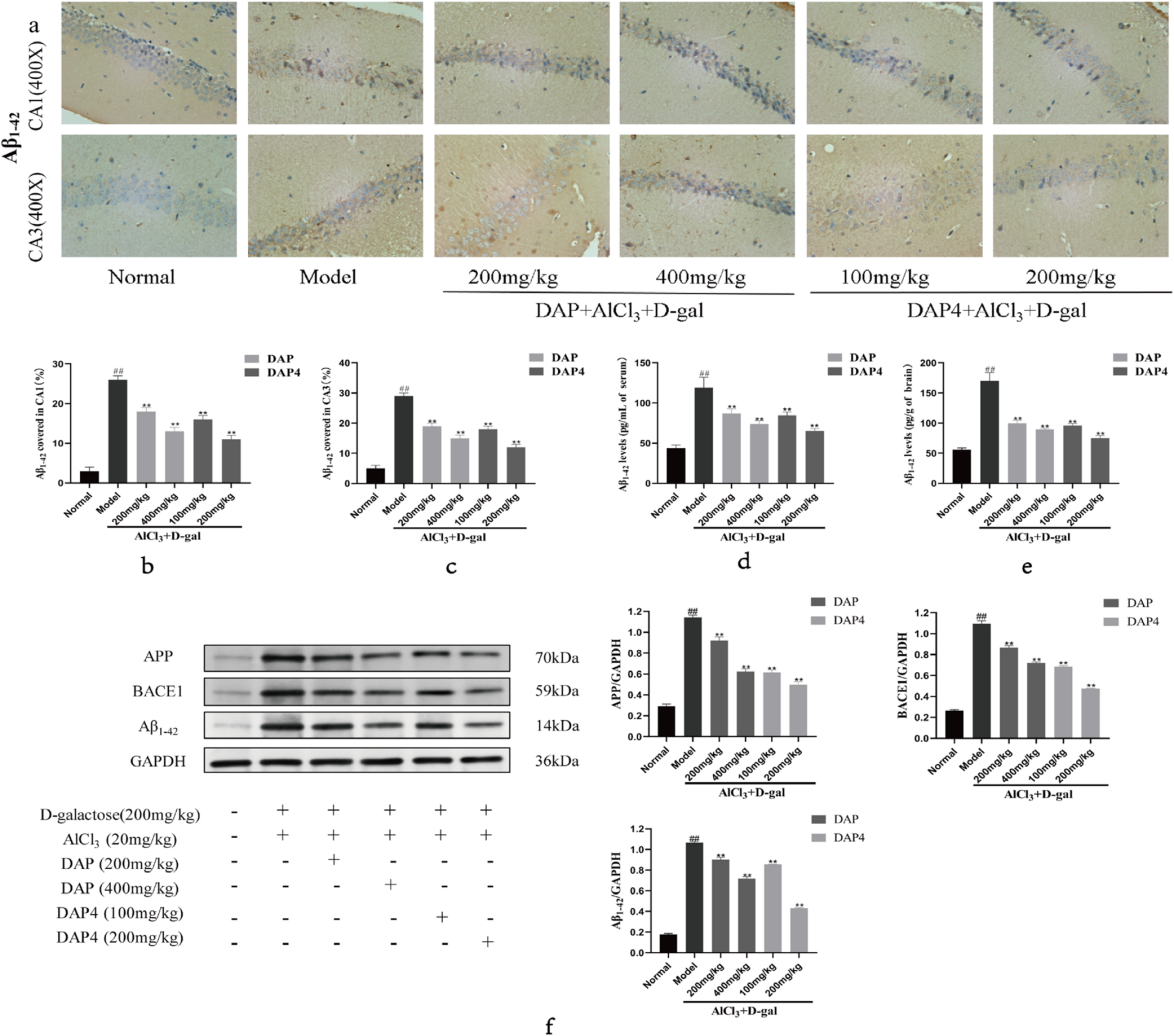
Effects of DAP and DAP4 on Aβ_1‐42_ in AlCl_3_/D‐gal‐induced AD mice. ^#^ *p* < 0.05, ^##^ *p* < 0.01 compared with that of the normal group; *p < 0.05, **p < 0.01, compared with that of the model group. (a) Aβ_1–42_ immunohistochemical representative images. (b) The expression of Aβ_1–42_ in the CA1 region. (c) The expression of Aβ_1–42_ in the CA3 region. (d) Serum Aβ_1–42_ levels. (e) Brain tissue Aβ_1–42_ levels. (f) APP, BACE1, and Aβ_1–42_ protein expression.

Aggregation of Aβ to form senile plaques and hyperphosphorylation of Tau proteins to form neurofibrillary tangles (NFT) are two major hallmarks of AD. As shown in Figure 4a, DAP and DAP4 significantly reduced yellowish‐brown Aβ plaque deposition and reduced CA1 (Figure 4b) and CA3 (Figure 4c) plaque deposition compared with that in the AlCl_3_/D‐gal model group. As shown in Figure 4d–e, ELISA analysis revealed that Aβ_1‐42_ levels in serum and brain tissues were notably decreased by DAP and DAP4 compared with that in the AlCl_3_/D‐gal model group. The findings indicate that DAP and DAP4 are capable of decreasing Aβ generation in blood and brain samples, as well as preventing the development of age spots in mice.

Beta‐secretase 1 (BACE1) is an important enzyme that plays a crucial role in breaking down amyloid precursor protein (APP) to produce Aβ. Western blot analysis was used to detect the APP, BACE1, and Aβ_1–42_ levels (Figure 4f). A significant increase in APP, BACE1, and Aβ_1‐42_ levels was observed in the AlCl_3_/D‐gal model group compared with that in the control group. In contrast, administration of DAP and DAP4 significantly decreased APP, BACE1, and Aβ_1‐42_ levels in the AlCl_3_/D‐gal model group. Hence, DAP and DAP4 were able to block the breakdown of APP into Aβ by suppressing the expression of BACE1.

**FIGURE 5:**
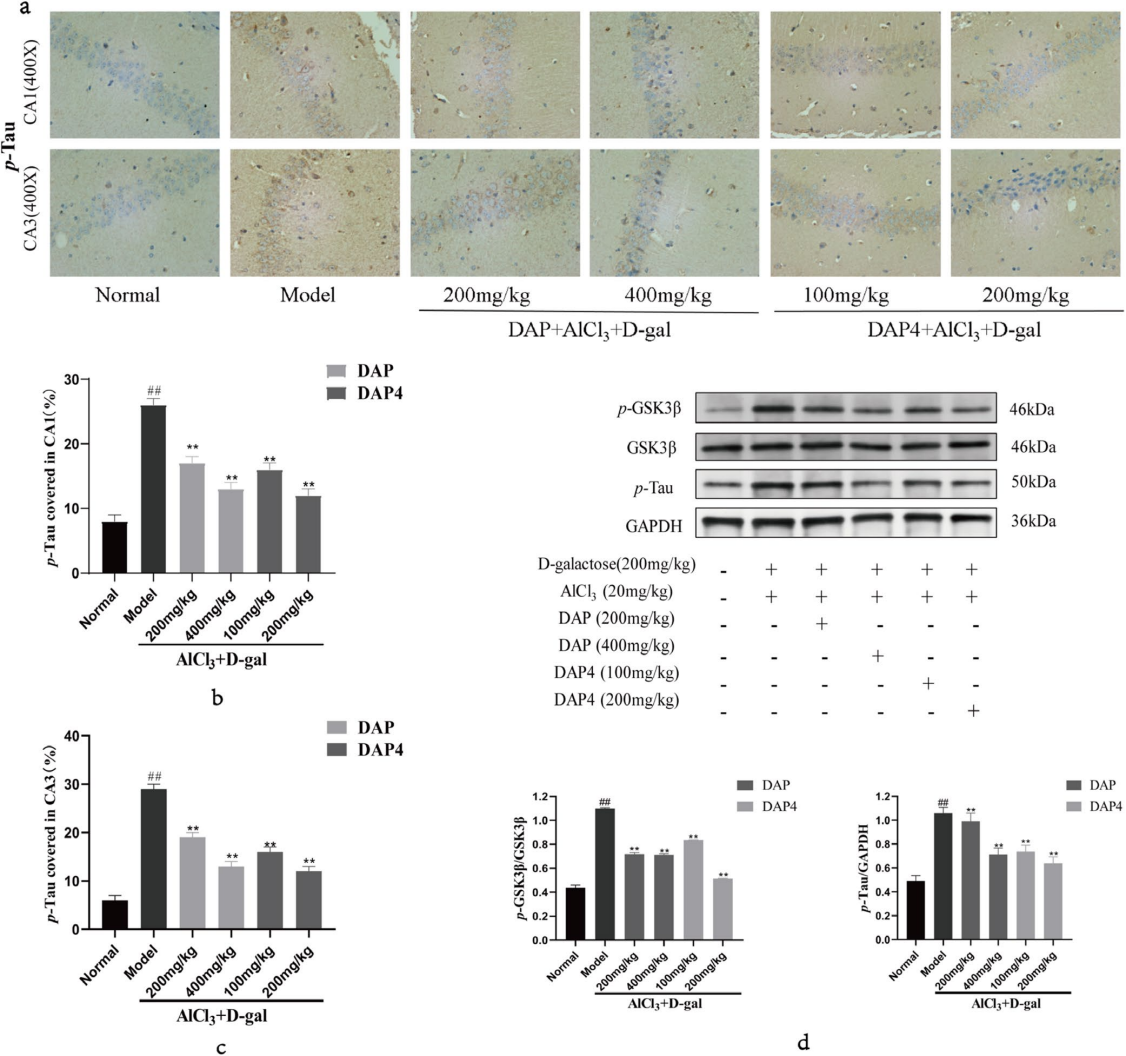
Effects of DAP and DAP4 on Aβ_1–42_ and *p‐*Tau in AlCl_3_/D‐gal‐induced AD mice. ^#^ *p* < 0.05, ^##^ *p* < 0.01 compared with that of the normal group; *p < 0.05, **p < 0.01, compared with that of the model group. (a) *p‐*Tau immunohistochemical representative images. (b) The expression of *p‐*Tau in the CA1 region. (c) The expression of *p‐*Tau in the CA3 region. (d) GSK3β, *p‐*GSK3β, and *p‐*Tau protein expression.

Tau acts as a microtubule‐associated protein that stabilizes the neuronal cytoskeleton, and its overphosphorylation results in neurogenic fiber tangle formation. As shown in Figure 5a, both DAP and DAP4 suppressed Tau phosphorylation and notably reduced *p*‐Tau aggregation in the CA1 (Figure 5b) and CA3 (Figure 5c) areas compared with that in the AlCl_3_/D‐gal model group. Glycogen synthase kinase‐3 beta (GSK3β) is crucial in the expression of *p*‐Tau and facilitates the hyperphosphorylation of Tau proteins. GSK3β, *p*‐GSK3β, and *p*‐Tau expression were detected using western blot, and the results are shown in Figure 5d. In comparison with that of the control group, the AlCl_3_/D‐gal model group elevated the *p*‐GSK3β/GSK3β ratio and boosted the expression of *p*‐Tau protein. DAP and DAP4 administration significantly decreased the ratio of *p*‐GSK3β/GSK3β and downregulated *p*‐Tau protein expression compared with that in the AlCl_3_/D‐gal model group. The findings indicated that DAP and DAP4 were able to lessen Tau hyperphosphorylation and diminish NFTs by inhibiting the activity of *p*‐GSK3β.

### 3.5 Effects of DAP and DAP4 on Oxidative Stress in AD Mice

**FIGURE 6:**
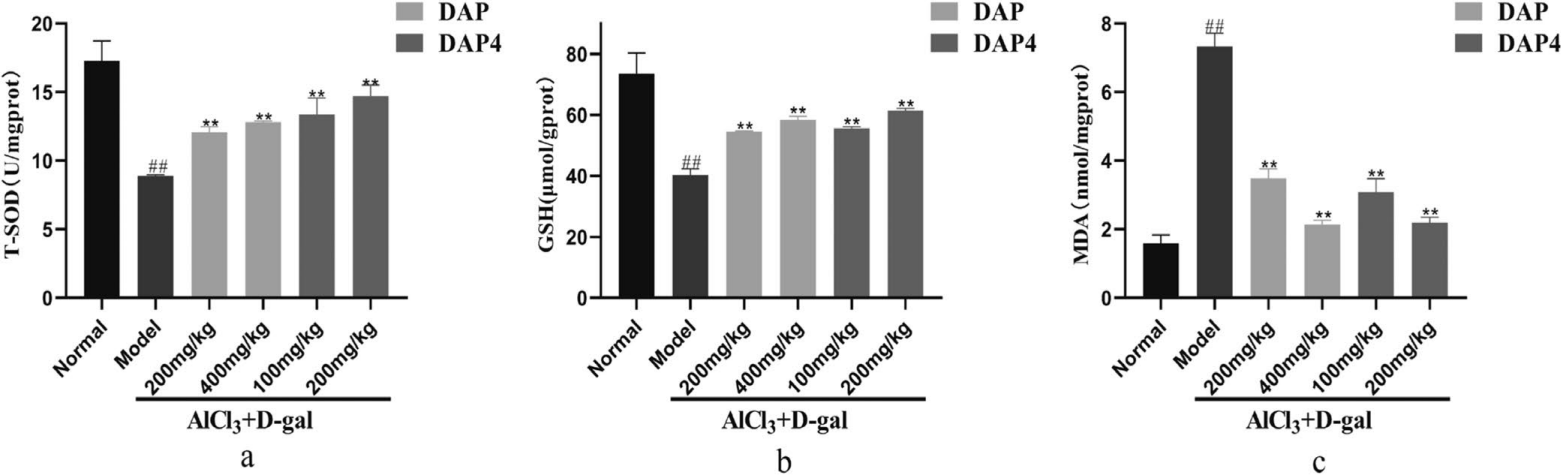
The effects of DAP and DAP4 on oxidative stress levels in AlCl_3_/D‐gal‐induced AD mice. ^#^ *p* < 0.05, ^##^ *p* < 0.01 compared with that in the normal group; *p < 0.05, **p < 0.01, compared with that in the model group. (a) T‐SOD activity in brain tissue. (b) GSH content in the brain tissue. (c) MDA content in the brain tissue.

The relationship between oxidative stress and AD biological processes was examined by measuring the T‐SOD, MDA, and GSH levels in mouse brains, as shown in Figure 6. Compared with that in the control group, the AlCl_3_/D‐gal model group showed a notable decrease in T‐SOD activity and GSH content along with a significant increase in MDA content. Compared with that in the AlCl_3_/D‐gal model group, a notable increase in T‐SOD activity (Figure 6a) and GSH content (Figure 6b) and a significant decrease in MDA content (Figure 6c) were observed after DAP and DAP4 administration. These findings indicated that DAP and DAP4 administration suppressed oxidative stress in the brains of mice with AD.

### 3.6 Effects of DAP and DAP4 on Neurochemical Indices in AD Mice

**FIGURE 7:**
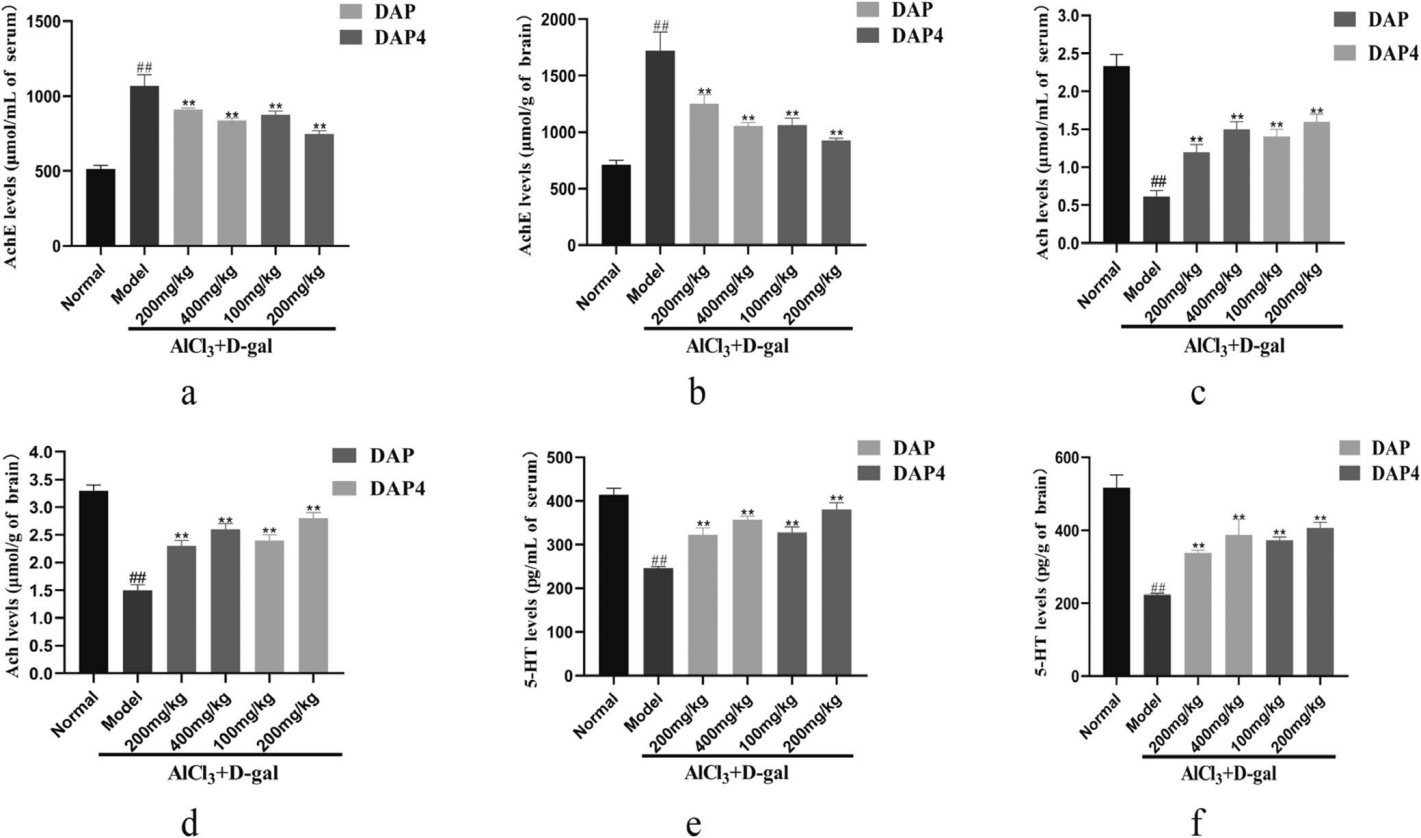
The effects of DAP and DAP4 on choline function in AlCl_3_/D‐gal‐induced AD mice. ^#^ *p* < 0.05, ^##^ *p* < 0.01 compared with that of the normal group; *p < 0.05, **p < 0.01, compared with that of the model group. (a) Serum AChE activity. (b) AChE activity in the brain tissue. (c) Serum ACh levels. (d) ACh levels in brain tissue. (e) Serum 5‐HT levels. (f) 5‐HT levels in brain tissue.

Figure 7 demonstrates that the AlCl_3_/D‐gal model group had notably boosted AChE activity and notably reduced ACh and 5‐HT concentrations in both serum and brain tissues when compared with that in the control group. DAP and DAP4 significantly decreased AChE activity (Figure 7a,b) and increased ACh (Figure 7c,d) and 5‐HT (Figure 7e,f) concentrations in the serum and brain tissue compared with that in the AlCl_3_/D‐gal model group. These findings indicated that the use of DAP and DAP4 reduced the negative effects of AlCl_3_/D‐gal on cholinergic function and enhanced the production of neurotransmitters.

### 3.7 Effects of DAP and DAP4 on Inflammation in the Brain of AD Mice

**FIGURE 8:**
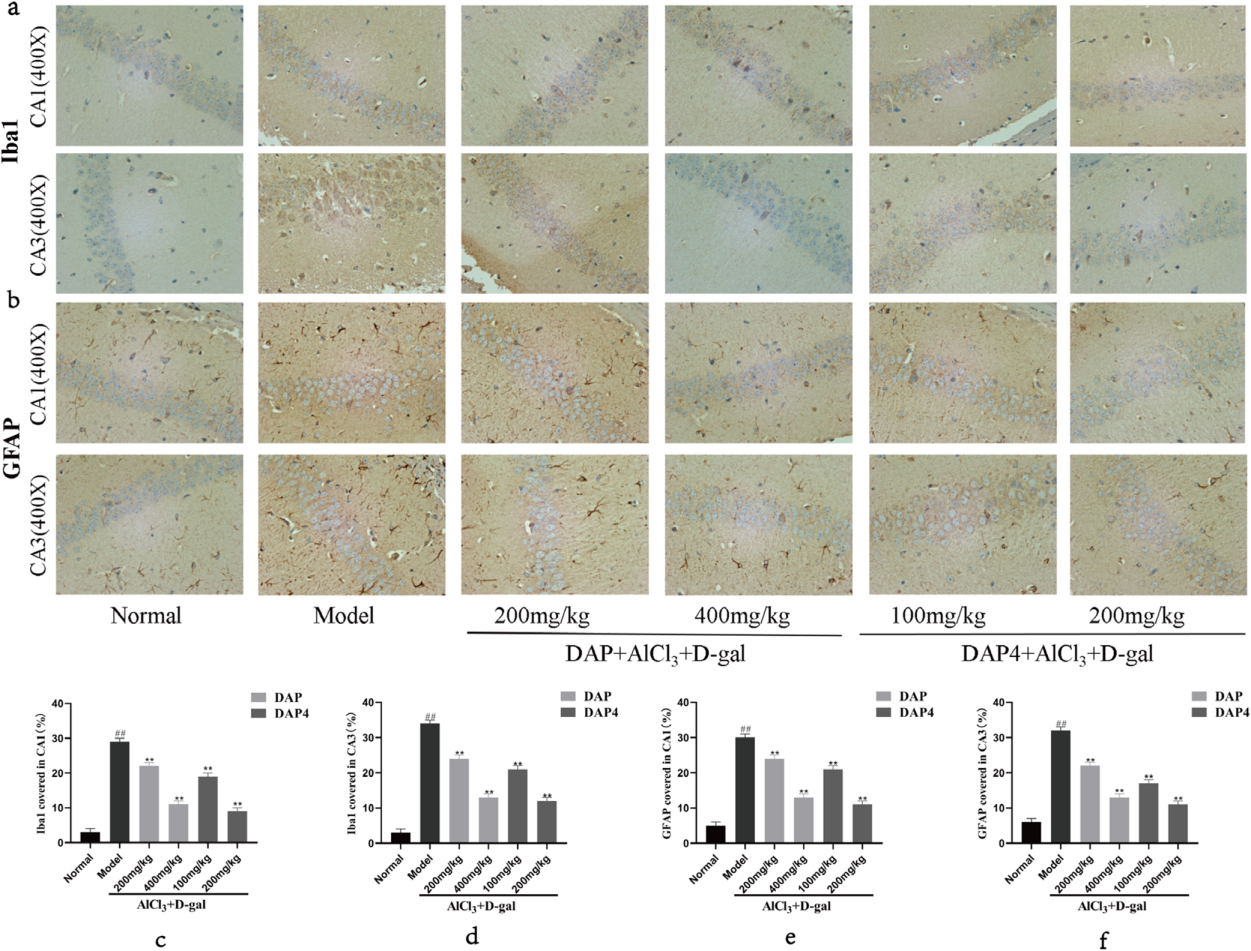
Effects of DAP and DAP4 on the expression of Iba1 and GFAP in the CA1 and CA3 regions of the hippocampus of AlCl3/D‐gal‐induced AD mice. ^#^ *p* < 0.05, ^##^ *p* < 0.01 compared with that in the normal group; *p < 0.05, **p < 0.01, compared with that in the model group. (a) Iba1 immunohistochemical representative images. (b) GFAP immunohistochemical representative images. (c) The expression of Iba1 in CA1. (d) The expression of Iba1 in the CA3 region. (e) The expression of GFAP in CA1. (f) The expression of GFAP in the CA3 region.

Neuroinflammation involves Iba1, GFAP, and neurons, and may result in damage to neurons and synapses, ultimately impairing memory and cognitive functions. Figure 8 illustrates that both DAP and DAP4 notably decreased the areas positive for Iba1 and GFAP in the CA1 (Figure 8a,c,d) and CA3 (Figure 8b,e,f) regions and reduced the enrichment of Iba1 and GFAP compared with that in the AlCl_3_/D‐gal model group. These findings indicate that DAP and DAP4 could potentially protect the brains of AD mice by preventing glial cell activation induced by AlCl_3_/D‐gal.

### 3.8 Effects of DAP and DAP4 on Apoptosis in AD Mice

**FIGURE 9:**
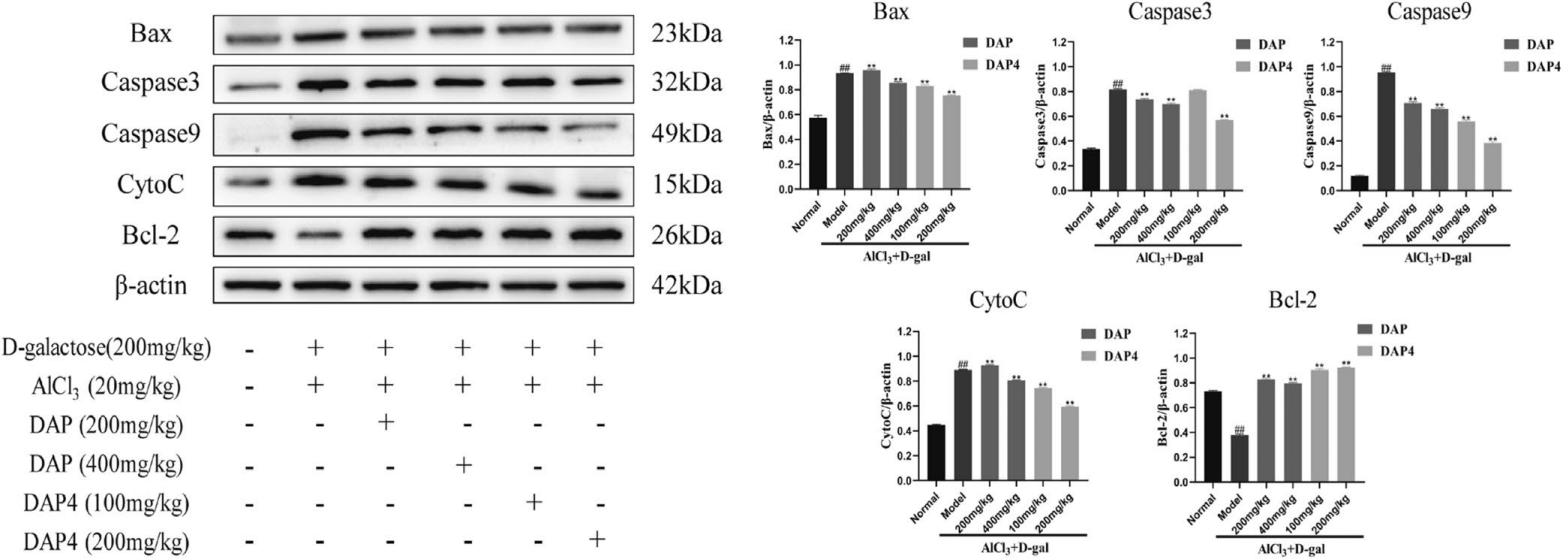
The effects of DAP and DAP4 on the expression of apoptosis‐related factors in AlCl_3_/D‐gal‐induced AD mice. ^#^ *p* < 0.05, ^##^ *p* < 0.01 compared with that of the normal group; *p < 0.05, **p < 0.01, compared with that of the model group.

Apoptosis is a process by which cells die in an autonomous and orderly manner under normal conditions through gene regulation. Figure 9 demonstrates that the AlCl_3_/D‐gal model group notably increased the expression of Caspase 3, Caspase 9, Bax, and CytoC proteins while decreasing the expression of Bcl‐2 protein, compared with that in the control group. DAP and DAP4 administration significantly downregulated Caspase 3, Caspase 9, Bax, and CytoC protein expression and upregulated Bcl‐2 protein expression compared with that of the AlCl_3_/D‐gal model group. These results suggest that DAP and DAP4 administration reduced AlCl_3_/D‐gal‐induced apoptosis in the brain tissue of AD mice.

### 3.9 Regulation of PI3K/AKT/Nrf2 Signaling Pathway by DAP and DAP4

**FIGURE 10:**
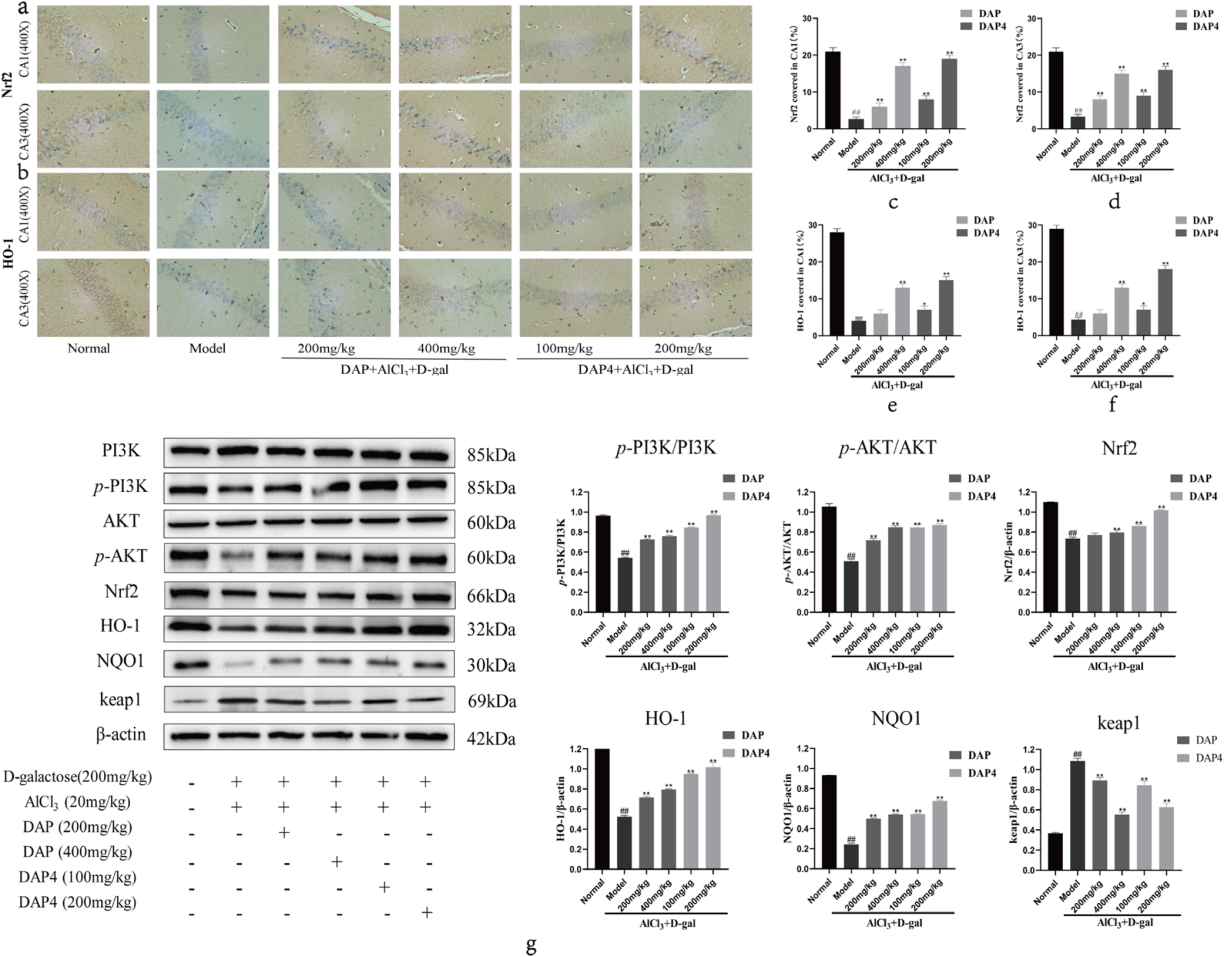
The effects of DAP and DAP4 on the PI3K/AKT/Nrf2 signaling pathway in AlCl_3_/D‐gal‐induced AD mice. ^#^ *p* < 0.05, ^##^ *p* < 0.01 compared with that of the normal group; *p < 0.05, **p < 0.01, compared with that of the model group. (a) Nrf2 immunohistochemical representative images. (b) HO‐1 immunohistochemical representative images. (c) Nrf2 expression in the CA1 region. (d) Nrf2 expression in the CA3 region. (e) HO‐1 expression in the CA1 region. (f) HO‐1 expression in the CA3 region. (g) Expression of PI3K/AKT/Nrf2 pathway‐related proteins.

The effects of DAP and DAP4 on the expression of proteins related to the PI3K/AKT and Nrf2 signaling pathways in the brain tissues of AlCl_3_/D‐gal‐induced AD mice were detected by western blot. As shown in Figure 10, immunohistochemical staining revealed that the AlCl_3_/D‐gal model group showed significantly downregulated Nrf2 and HO‐1 expression in the CA1 and CA3 regions compared with that in the control group. DAP and DAP4 administration significantly upregulated Nrf2 (Figure 10a,c,d) and HO‐1 (Figure 10b,e,f) expression in the CA1 and CA3 regions compared with that in the AlCl_3_/D‐gal model group mice. Western blot analysis showed that the AlCl_3_/D‐gal model significantly downregulated Nrf2, HO‐1, NQO1, *p*‐PI3K, and *p*‐AKT protein expression and significantly upregulated keap1 protein expression compared with that in the control group. As shown in Figure 10g, DAP and DAP4 administration significantly upregulated Nrf2, HO‐1, NQO1, *p*‐PI3K, and *p*‐AKT protein expression and significantly downregulated keap1 protein expression compared with that in the AlCl_3_/D‐gal model group.

### 3.10 Effects of DAP and DAP4 on the Intestinal Flora of AD Mice

**FIGURE 11:**
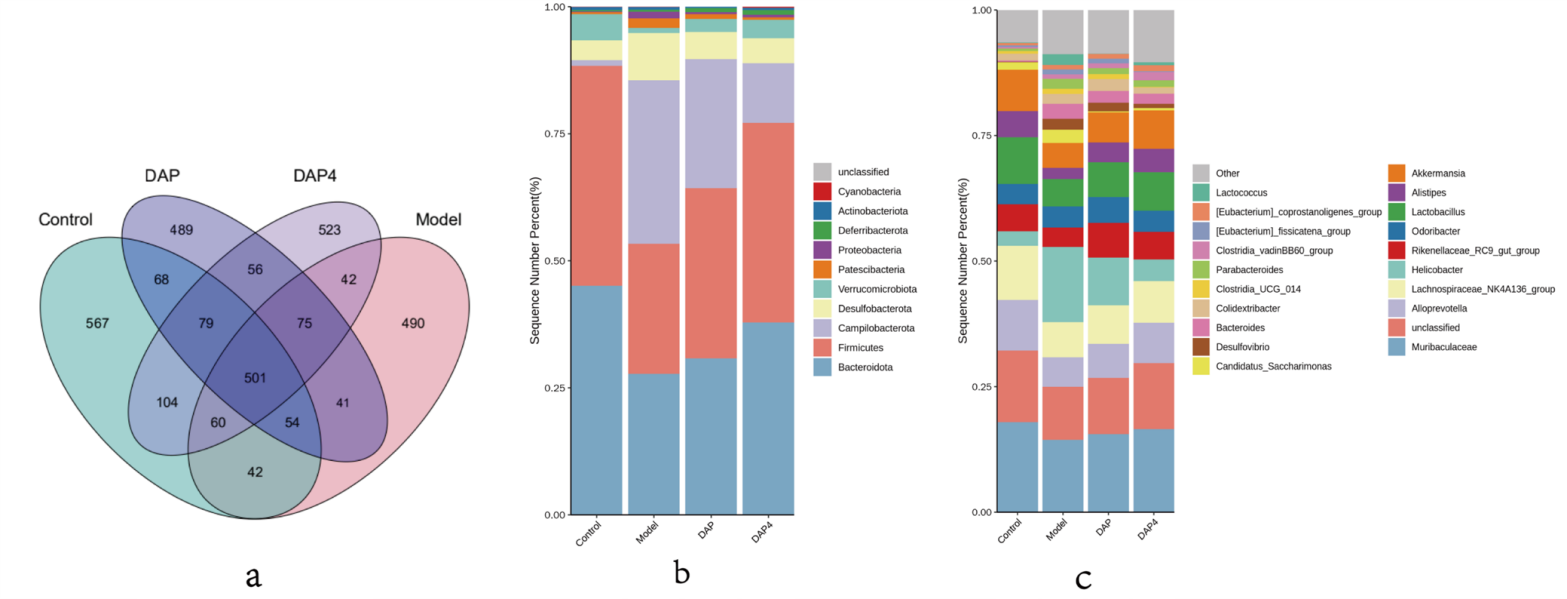
Comparison of changes in intestinal flora in different groups. (a) Venn diagram analysis of common and endemic species. (b) Bar chart showing the relative distribution of individual samples at the phylum level. (c) Histogram of the relative distribution of individual samples at the genus level.

Increasing evidence suggests that gut microbes play an important role in the development of AD. The effects of sika deer antler protein on the gut flora of AD mice were assessed using 16S rRNA gene sequencing. The Venn diagram shows the number of operational taxonomic units (OTU) in the corresponding subgroups, specific relationships for each subgroup, and shared OTUs, as shown in Figure 11a, with a total of 3191 OTUs. There were 1475 OTUs in the control group, 1305 in the AD model group, 1363 in the DAP group, and 1440 in the DAP4 group, suggesting that DAP and DAP4 play a role in maintaining the abundance of gut microbes. Subsequently, the four gut microbial structure groups were assessed at different levels. As shown in Figure 11b, the composition of bacterial groups at the phylum level and the analysis of differential species showed that each group was mainly dominated by Firmicutes, Bacteroidota, Verrucomicrobiota, and Proteobacteria at the phylum level. Administration of DAP and DAP4 increased the abundance of Bacteroidota and Campilobacterota dominant bacteria and decreased the abundance of Campilobacterota and Desulfobacterota pathogenic bacteria. As shown in Figure 11c, the groups were dominated by *Muribaculaceae* and *Lachnospiraceae* at the family level and *Alloprevotella, Lactobacillus, Alistipes*, and *Akkermansia* at the genus level. DAP and DAP4 increased the abundance of beneficial bacteria, such as *Muribaculaceae, Alloprevotella, Lachnospiraceae_NK4A136_group, Lactobacillus*, and *Alistipes*, and decreased the abundance of harmful bacteria, such as *Helicobacter* and *Desulfovibrio*.

**FIGURE 12:**
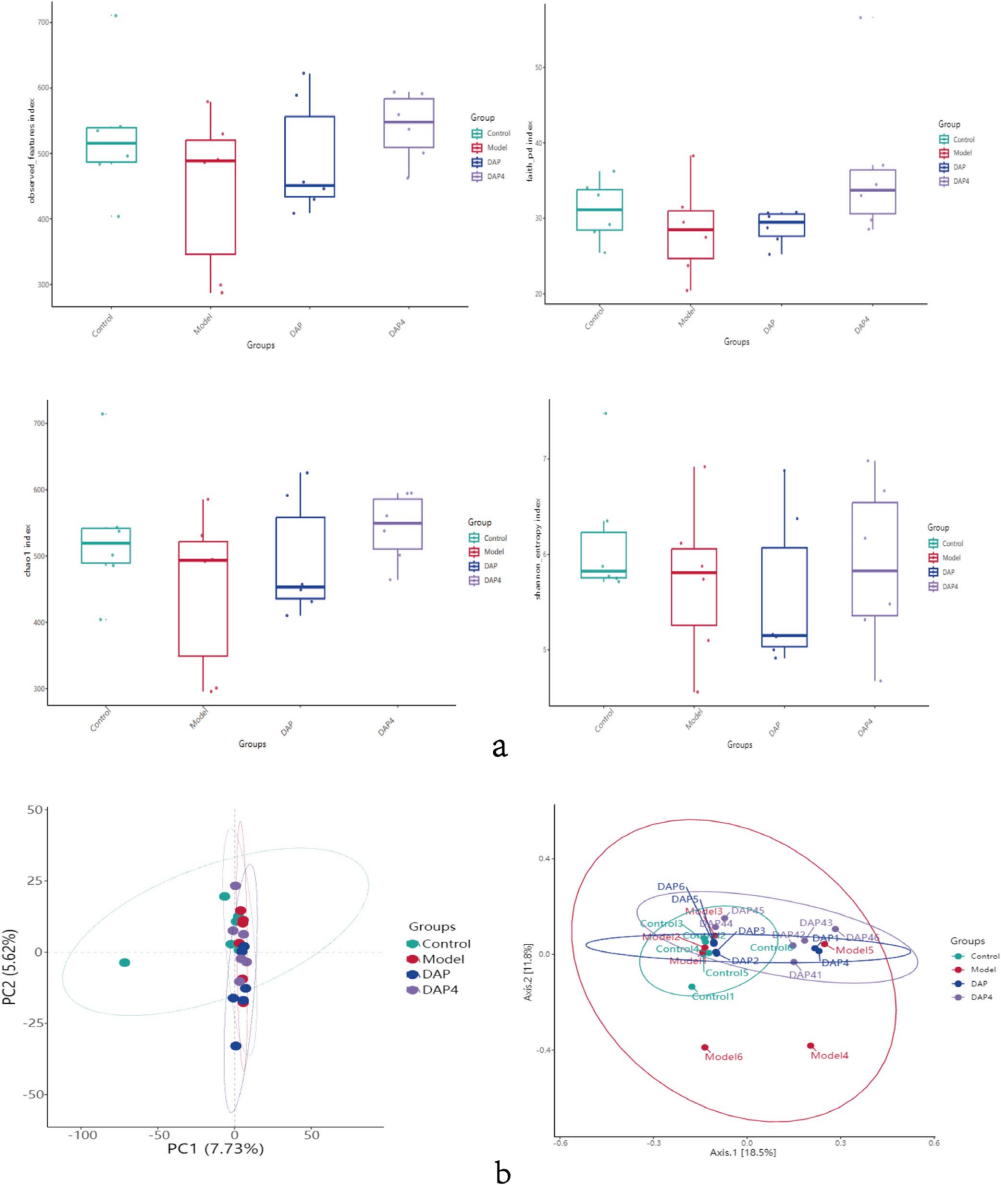
Analysis of α‐diversity index and β‐diversity indices. (a) Boxplot of α‐diversity indices for Chao1, faith_pd, Observed_features, and Shannon indices. (b) The β‐diversity analysis with PCA and PCoA distribution plots, respectively.

The α‐diversity index and β‐diversity index are commonly used to assess the diversity of microorganisms. The Shannon sparse curves of each group stabilized with an increase in read sequences, indicating that the sample sequencing data were reasonable and reflected the community structure of the sample. The Chao1, faith_pd, and Observed_features indices were used to assess the sample species richness, and the Shannon index was used to assess the microbial community diversity, as shown in Figure 12a, where DAP and DAP4 administration increased the flora richness and diversity. The β‐diversity was primarily used to assess differences in the gut microbe structure and composition. As shown in Figure 12b, the samples in the same groups were in close proximity and no samples had significant outliers.

**FIGURE 13:**
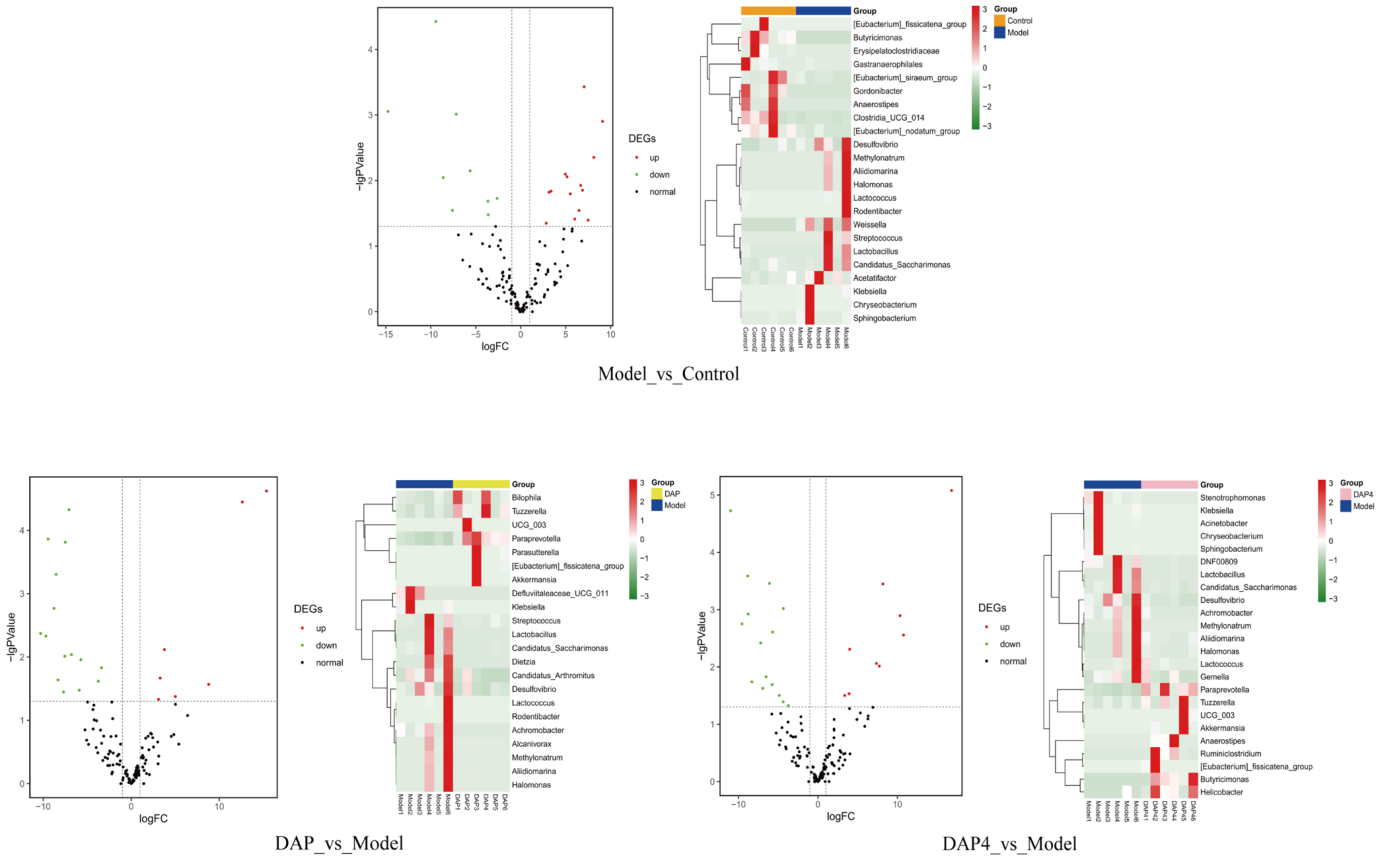
Volcano plot and heat map analysis of significantly differentially distributed colonies. Left: Test volcano plot, red and green dots indicate significantly upregulated and downregulated expression colonies, horizontal dashed lines indicate *p* < 0.05; Right: Heat map of gene clustering based on expression levels of significantly different colonies.

Screening and analysis of the intestinal flora showed that 26 flora were significantly different in the model group compared with those in the control group, of which 14 were upregulated and nine were downregulated. Compared with that in the model group, 22 colonies were significantly different in the DAP group, with seven colonies upregulated and 15 colonies downregulated, whereas 24 colonies were significantly different in DAP4, with nine colonies upregulated and 15 colonies downregulated. The test volcano plots and heatmaps based on the level of abundance of the differing colonies in each group are shown in Figure 13.

### 3.11 Effects of DAP and DAP4 on Metabolites in Brain and Intestinal Tissues of AD Mice

**FIGURE 14:**
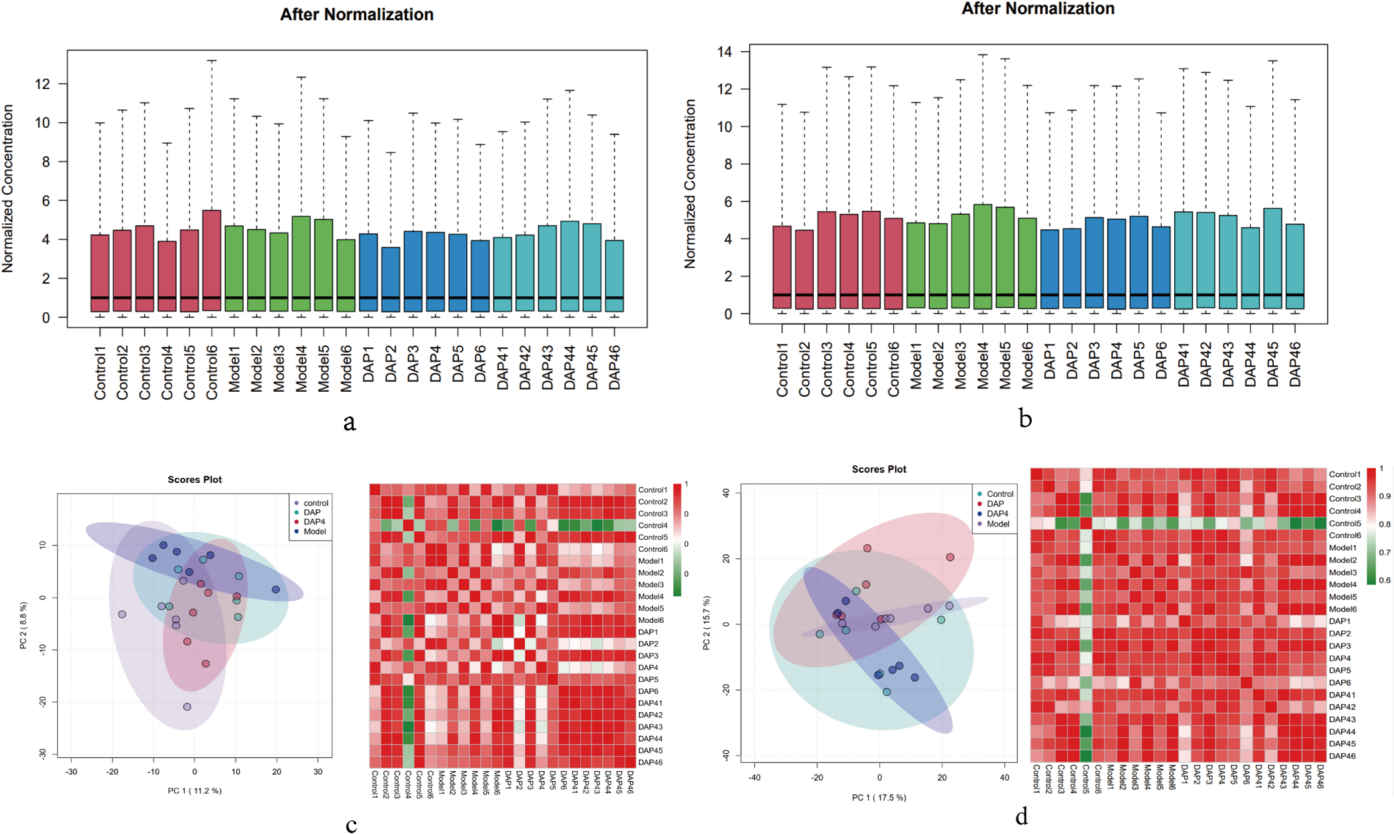
Metabolome analysis of different groups. (a) Distribution of metabolites in the brain tissue. (b) Metabolite distribution maps of intestinal tissues. (c) PCA distribution map and sample correlation heat map of brain tissue based on metabolite abundance. (d) PCA distribution map and sample correlation heat map of intestinal tissue based on metabolite abundance.

To investigate the neuroprotective mechanisms of DAP and DAP4, brain and intestinal tissue metabolic analyses were performed using UPLC‐Q‐Exactive‐mass spectrometry. The data were first extracted and normalized to obtain metabolite abundance data, as shown in Figure 14a,b. The median values of the data were almost on a horizontal line, indicating that the expression profiling data were well standardized and could be used directly. Based on the expression abundance of each sample, multiple methods were utilized to assess the correlation between the samples, which were first subjected to PCA clustering analysis based on expression abundance. The results are shown in Figure 14c,d. Overall, the distribution of all samples was more concentrated, indicating that there was little difference between the samples, the overall performance of the samples was more desirable, and no outlier samples were present. The Pearson correlation coefficient between each pair of samples was then calculated, and the closer the coefficient was to 1, the higher the similarity of the expression pattern between the samples, as shown in the sample correlation heat map. The correlation between all the samples was greater than 0.6, which indicates that the correlation between them was high.

**FIGURE 15:**
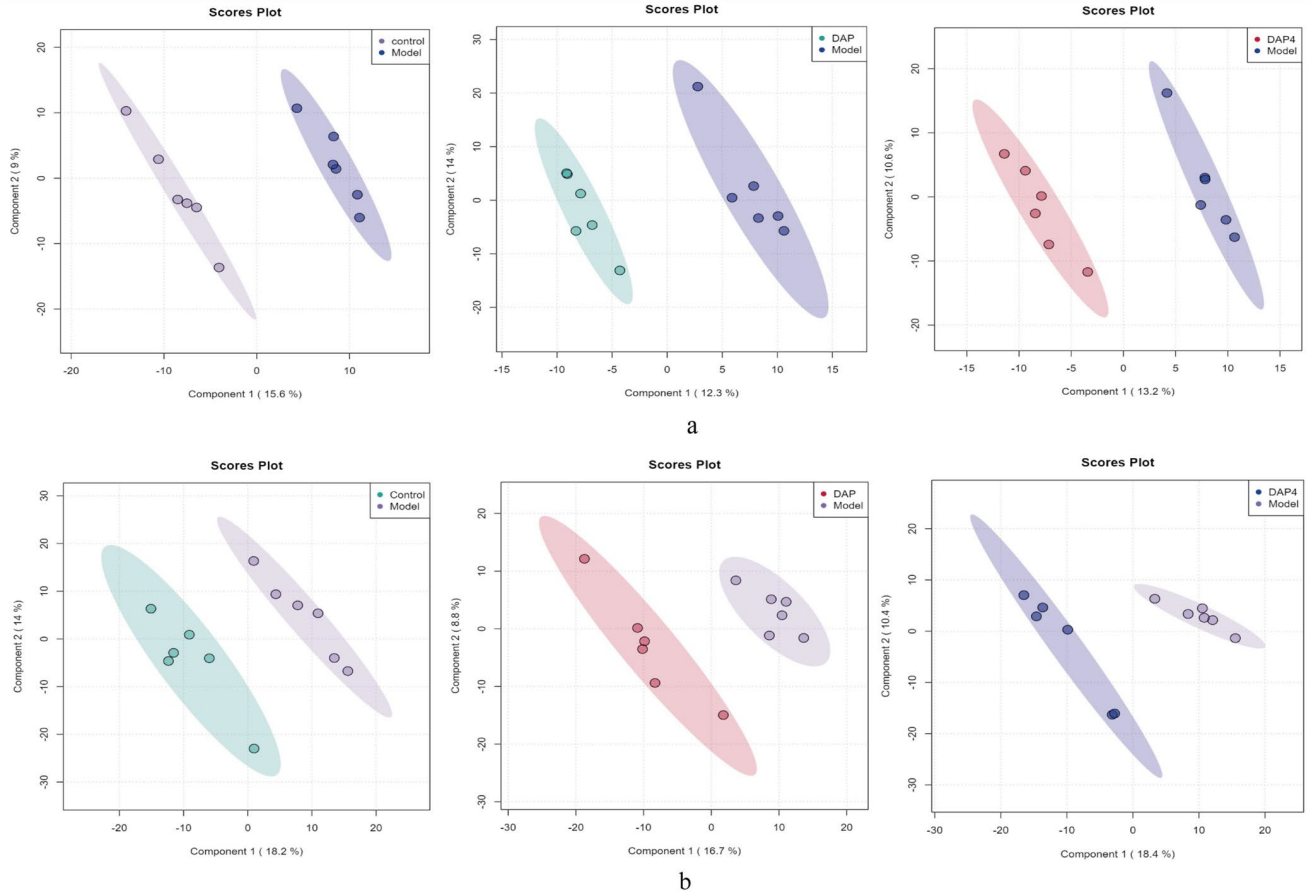
Metabolomics PLS‐DA plot. (a) PLS‐DA plot of brain tissue metabolomics. (b) PLS‐DA plot of the intestinal tissue metabolomics. In the PLS‐DA plot, each point corresponds to a sample, and the horizontal and vertical coordinates are the values of the two factors with the best discriminatory effect. Each grouping is indicated by a different color. The areas indicated within the ellipses are the 95% confidence intervals of the sample points: (left) Model_vs_Control, (middle) DAP_vs_Model, and (right) DAP4_vs_Model.

PLS‐DA searches for a linear regression model by projecting the predictor and observed variables separately in a new space. As shown in Figure 15a,b, two features with clear distinguishing effects were selected and plotted as scatter plots. If the data points of the samples from different groups are scattered across different regions, the PLS‐DA model is more effective in classifying the metabolites that are significantly different between the different groups. For the three sample groups, Model_ vs. _Control, DAP_ vs. _Model, and DAP4_ vs. _Model, the two sample point clouds in each group were distributed in different regions with large differences in distance. This suggests that there are significant differences in the metabolites between the subgroups, which warrant further analysis.

**FIGURE 16:**
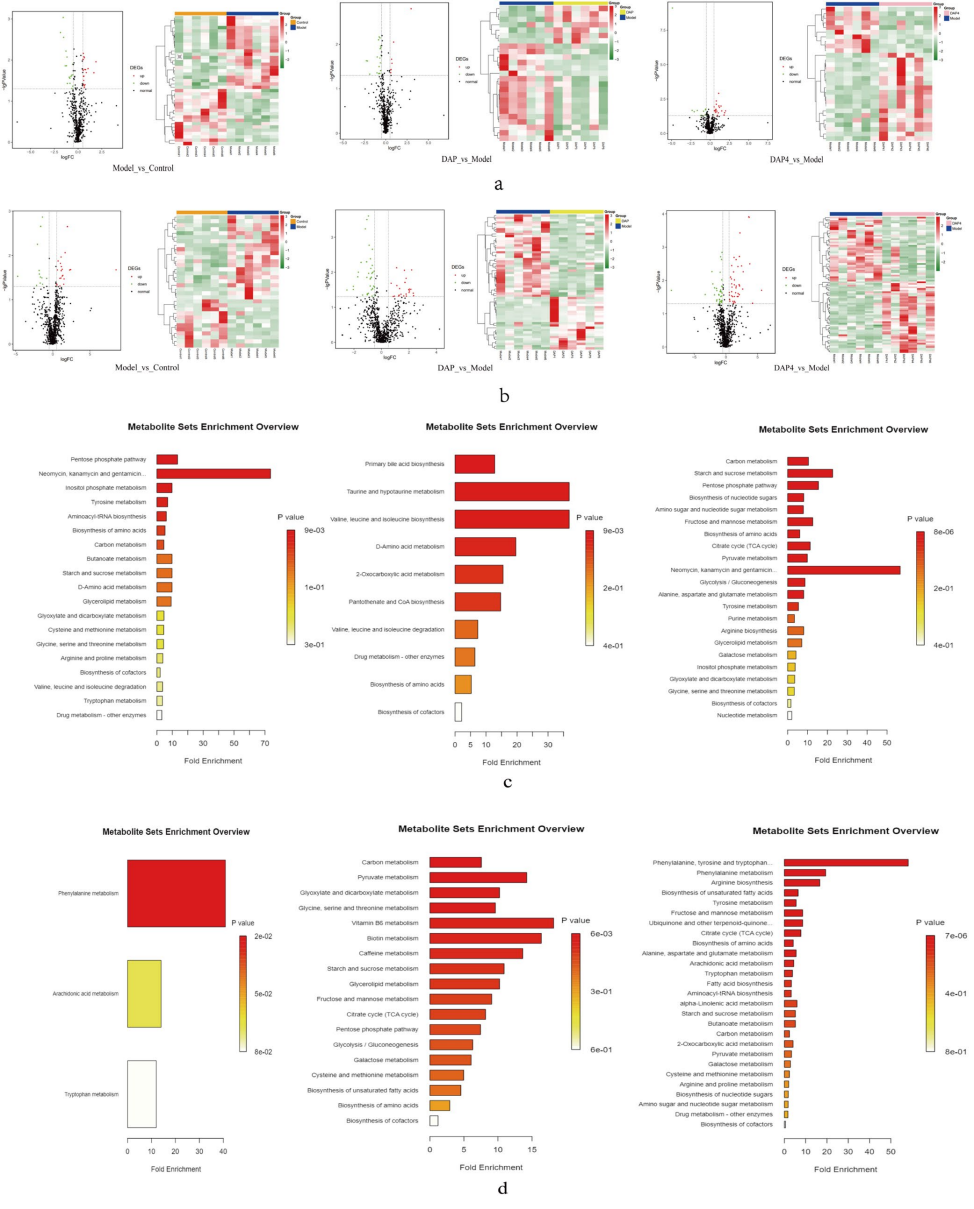
Volcanograms and thermograms of metabolomics differential metabolites. (a) Brain tissue metabolomics: Differential metabolite volcano plot and heat map. (b) Intestinal tissue metabolomics: Differential metabolite volcano and heat maps. (c) Brain tissue metabolomics: Comparison of differential metabolites and KEGG pathway enrichment analysis result plots. (d) KEGG pathway enrichment analysis of differential metabolites in the comparative group of intestinal tissue metabolomics. In the differential metabolite volcano plot analysis, red and green dots indicate significantly upregulated and downregulated metabolites, horizontal dashed lines indicate *p* < 0.05, and vertical dashed lines indicate |LogFC > 0.5, (left) Model_ vs. _Control, (center) DAP_ vs. _Model, (right) DAP4_ vs. _Model.

The metabolites that met the threshold |logFC| > 0.5, *p* < 0.05, and VIP ≥ 1.0 were selected as differential metabolites by screening for significantly differentially distributed metabolites in each of the three comparison groups. Figure 16a,b show the significantly differentially distributed metabolites obtained by screening in each of the three groups. The metabolites screened in the comparison group were then analyzed for KEGG metabolic signaling pathway enrichment, and the results are shown in Figure 16c,d. The metabolic pathways enriched in the brain tissue were mainly phenylalanine, tyrosine, and tryptophan biosynthesis; arginine biosynthesis; histidine metabolism; amino acid and nucleotide sugar metabolism; and other pathways. The metabolic pathways enriched in intestinal tissues included pyruvate metabolism, lipid metabolism, arginine and proline metabolism, nucleotide metabolism, and other pathways.

### 3.12 STEM Trend Analysis

The samples were divided into two groups based on the results of the differential metabolite analysis. The DAP group of differential metabolites was the concatenation of the Model vs. Control and DAP vs. Model groups of the differential metabolites. As shown by the analysis of differential metabolites in the brain tissue, 57 differential metabolites were obtained. Analysis of the intestinal tissue revealed 87 differential metabolites, and the differential metabolites in the DAP4 group were the concatenation of the differential metabolites in the Model_vs_Control and DAP4_vs_Model groups. As shown by the analysis of the brain groups, 61 differential metabolites were identified. A total of 106 differential metabolites were obtained by analyzing the intestinal tissue.

**FIGURE 17:**
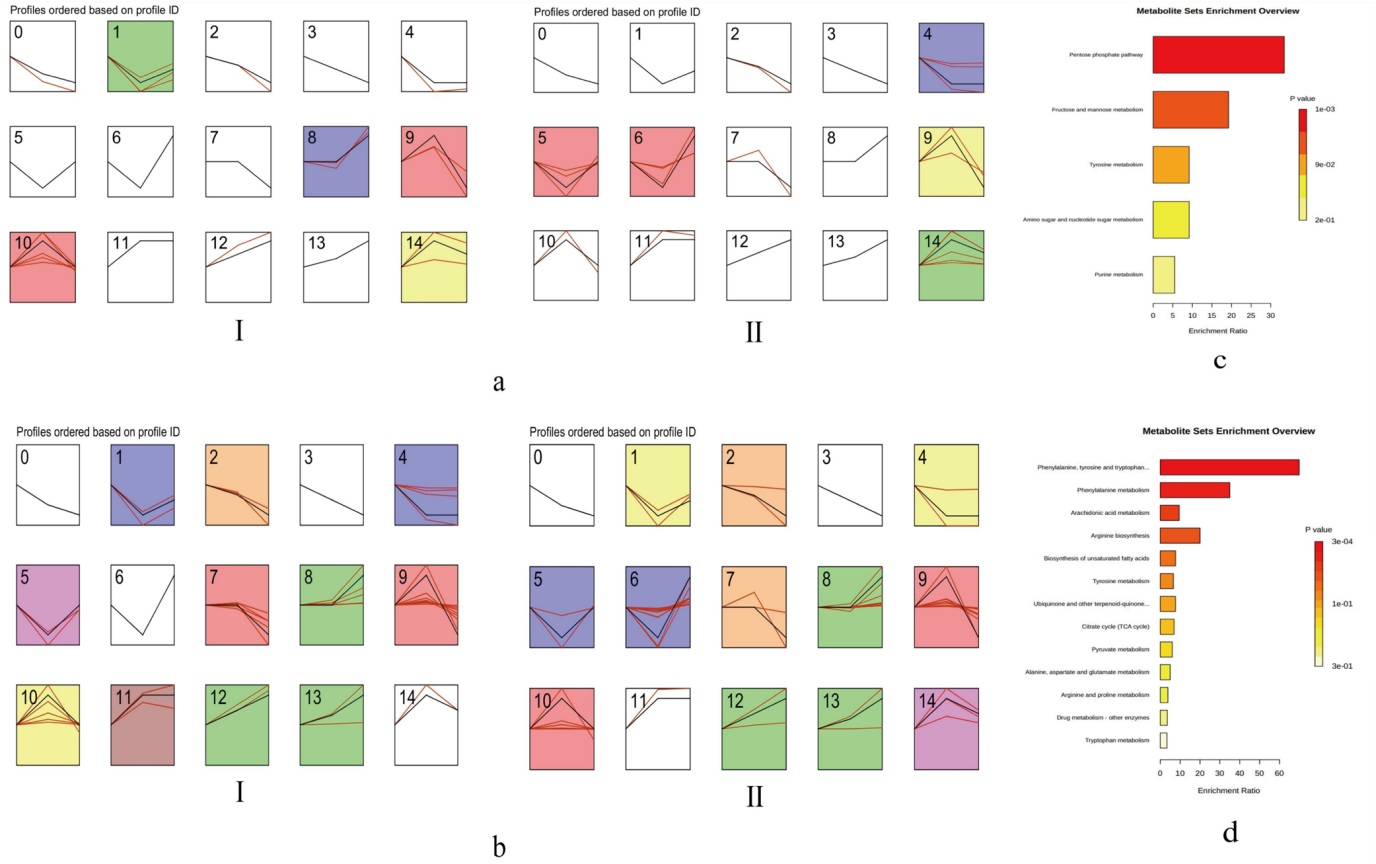
STEM analysis of differential metabolites in brain and intestinal tissues. (a) Results of STEM cluster analysis of differential metabolites in brain tissue. (b) Results of STEM clustering analysis of intestinal tissue differential metabolites. (c) KEGG pathway enrichment bar graph of the brain tissue differential metabolite trend module in the DAP4 group. (d) KEGG pathway enrichment bar graph of intestinal tissue differential metabolite trend module in DAP4 group. In the STEM analysis, the left (I) and right (II) graphs show the results of STEM cluster analysis of overlapping metabolites in the DAP and DAP4 groups, respectively.

Significantly different metabolites were screened in brain and intestinal tissues, and significant similarity clustering of metabolite abundance patterns was carried out using STEM software; clusters with different expression trends were screened, and the results are shown in Figure 17a,b. The main differential metabolites with trend changes obtained from brain tissues were analyzed as follows: oleoyl‐2‐palmitoyl‐RAC glycerol, 1‐stearoyl‐lysine‐glycerol, 8‐hydroxyquinoline‐2‐carbonitrile, docosahexaenoic acid‐1‐stearoyl‐SN‐glycerol‐3‐phosphate ethanolamine, 2,4,6‐tri‐tert‐butylaniline, eicosatetraenoic acid, thymosin β‐D‐glucoside anion, etc. The differential metabolites with trend changes obtained from the intestinal tissues were mainly tetrahydrobenzyl alcohol; 2‐amino‐2‐methyl‐1,3‐propanediol; 2‐bis[hydroxymethyl]‐2,2′,2′‐carbonitrile triethanol; 2,4‐diaminotoluene; 3‐hydroxybutanoic acid; arachidonic acid‐2′‐fluoroethylamide; D‐red‐imidazolylglycerophosphate; DL‐preadrenaline; and L‐palmitoylcarnitine.

In the clusters obtained from the screening, the following differential metabolites were investigated: those in the first increasing and then decreasing trend module and those in the first decreasing and then increasing trend modules. Differential metabolites with changing trends were analyzed separately using metaboanalyst 6.0. The DAP group differential metabolites had fewer enriched pathways when subjected to enrichment pathway analysis. Therefore, differentially expressed metabolites in the DAP4 group were selected for enrichment pathway analysis. KEGG pathway enrichment analysis was performed on the differential metabolites with changing trends in brain and intestinal tissues of the DAP4 group, and the pathways obtained by enrichment are shown in Figure 17c,d. The pentose phosphate pathway, fructose and mannose metabolism, tyrosine metabolism, amino sugar and nucleotide sugar metabolism, and urine metabolism were the main pathways of differential metabolite enrichment in brain tissue. Phenylalanine, tyrosine, and tryptophan biosynthesis; phenylalanine metabolism; arachidonic acid metabolism; arginine biosynthesis; biosynthesis of unsaturated fatty acids; and tyrosine metabolism were the main pathways of differential metabolite enrichment in the intestinal tissue.

### 3.13 Correlation Analysis of DAP4 on Gut Microbes and Differential Metabolites in AD Mice

**FIGURE 18:**
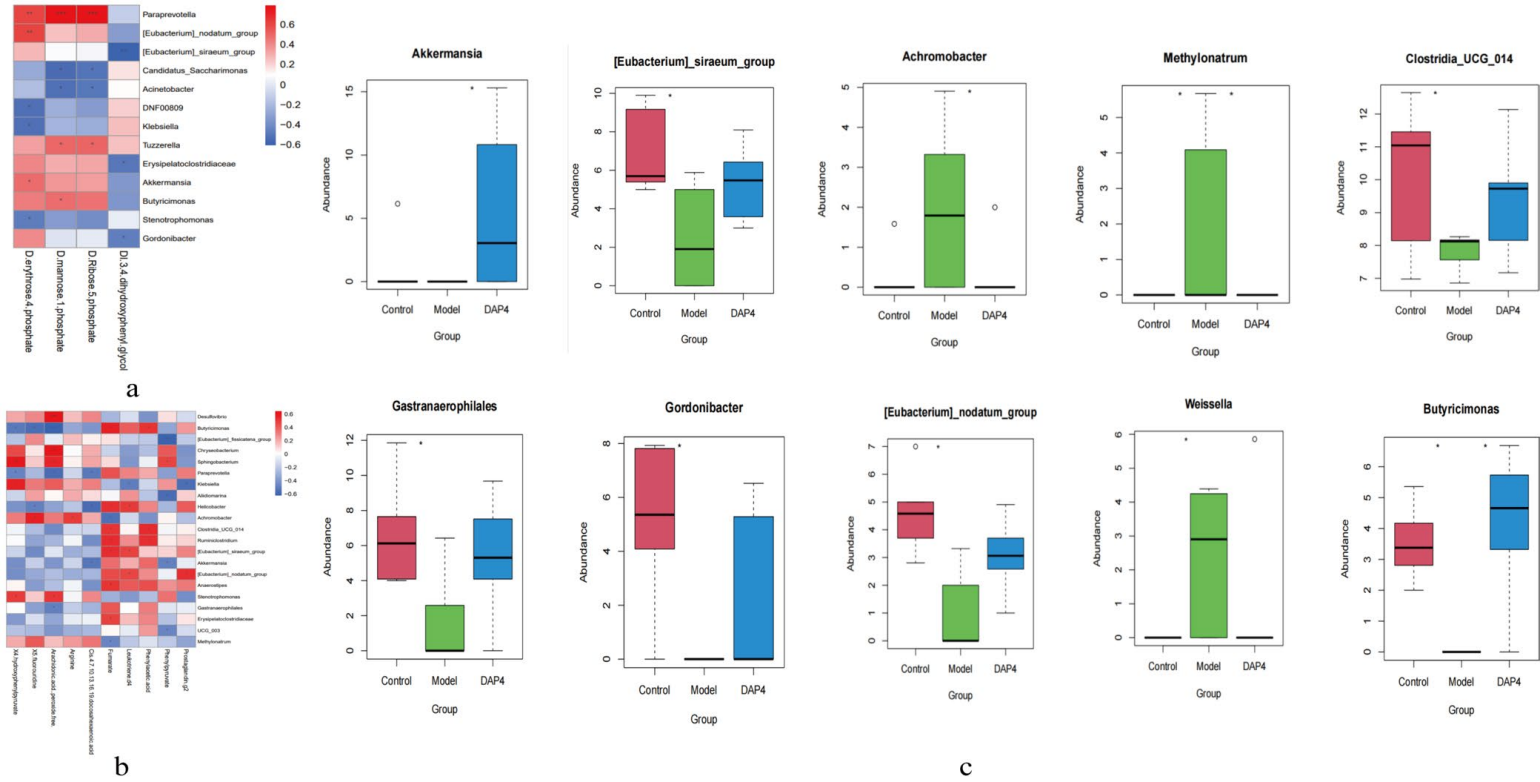
Correlation analysis of gut microbes and differential metabolites. (a) Heat map displaying the correlation between gut flora and brain tissue metabolite levels that satisfy the changing trend. (b) Heat map showing the correlation between the intestinal flora and intestinal tissue metabolite levels that satisfy the changing trend. (c) Intestinal flora that satisfy the change patterns of rising and then falling or falling and then rising.

The Spearman correlation was calculated between the intestinal flora obtained from DAP4 screening and the metabolites with trend differential distribution obtained from brain and intestinal tissue screening. The cor function in R4.3.1 was used, and *p* < 0.05 and absolute correlation coefficient values higher than 0.4 were selected as the screening threshold, and correlation heatmaps were constructed (Figure 18a,b). All pattern changes in bacterial groups obtained from DAP4 screening were examined, and the groups that satisfied the patterns of rising and then falling or falling and then rising in the normal disease medication process were retained. This yielded 10 groups that satisfied the changing trends (Figure 18c): *Akkermansia, [Eubacterium]_siraeum_ group, Achromobacter, Methylonatrum, Clostridia_UCG_014, Gastranaerophilales, Gordonibacter, [Eubacterium]_nodatum_group, Weissella*, and *Butyricimonas*.

**FIGURE 19:**
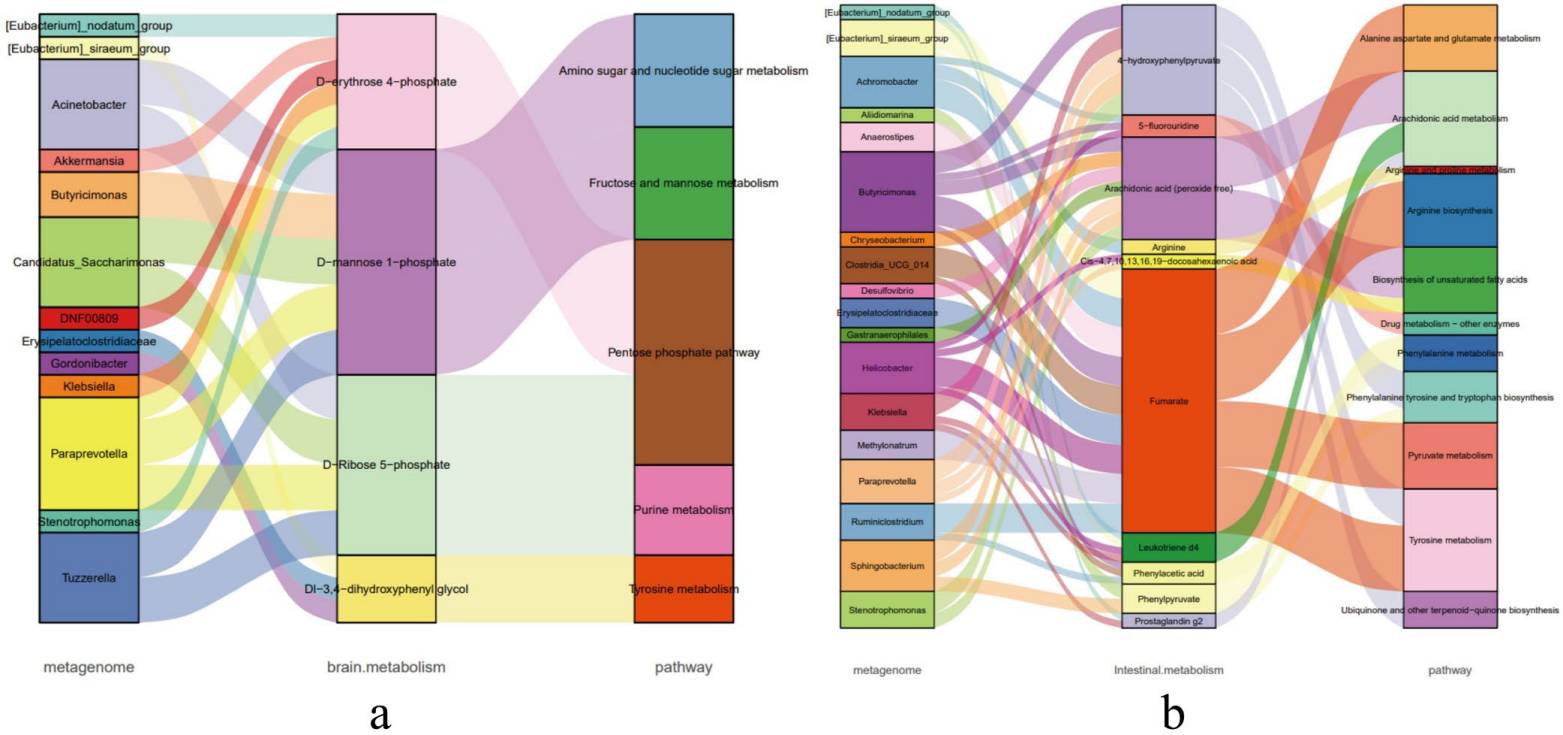
Sankey diagram for macrogenomic and metabolomic association analysis. (a) Sankey diagram of intestinal flora–brain tissue metabolome‐pathway regulatory routes. (b) Sankey diagram of intestinal flora–intestinal tissue metabolome pathway regulation routes.

Based on the above analyses, the association results of the macrogenome and metabolome were used to construct a Sankey diagram. The intestinal flora–brain tissue pathway regulation routes are shown in Figure 19a. The pathways that were jointly altered by the intestinal flora–brain tissue metabolites after DAP4 drug treatment were tyrosine metabolism, pentose phosphate pathway, fructose and mannose metabolism, amino sugar and nucleotide sugar metabolism, and urine metabolism. The intestinal flora–intestinal tissue pathway regulation routes are shown in Figure 19b, and the pathways that were co‐altered by DAP4 drug treatment, as shown by Sankey diagram analysis, were tyrosine metabolism, phenylalanine tyrosine and tryptophan biosynthesis, phenylalanine metabolism, arachidonic acid metabolism, arginine biosynthesis, biosynthesis of unsaturated fatty acids, ubiquinone and other terpenoid‐quinone biosynthesis, pyruvate metabolism, alanine aspartate and glutamate metabolism, arginine and proline metabolism, and drug metabolism‐other enzymes. The metabolic pathway co‐regulated by the brain and intestinal tissues was the tyrosine metabolic pathway.

**FIGURE 20:**
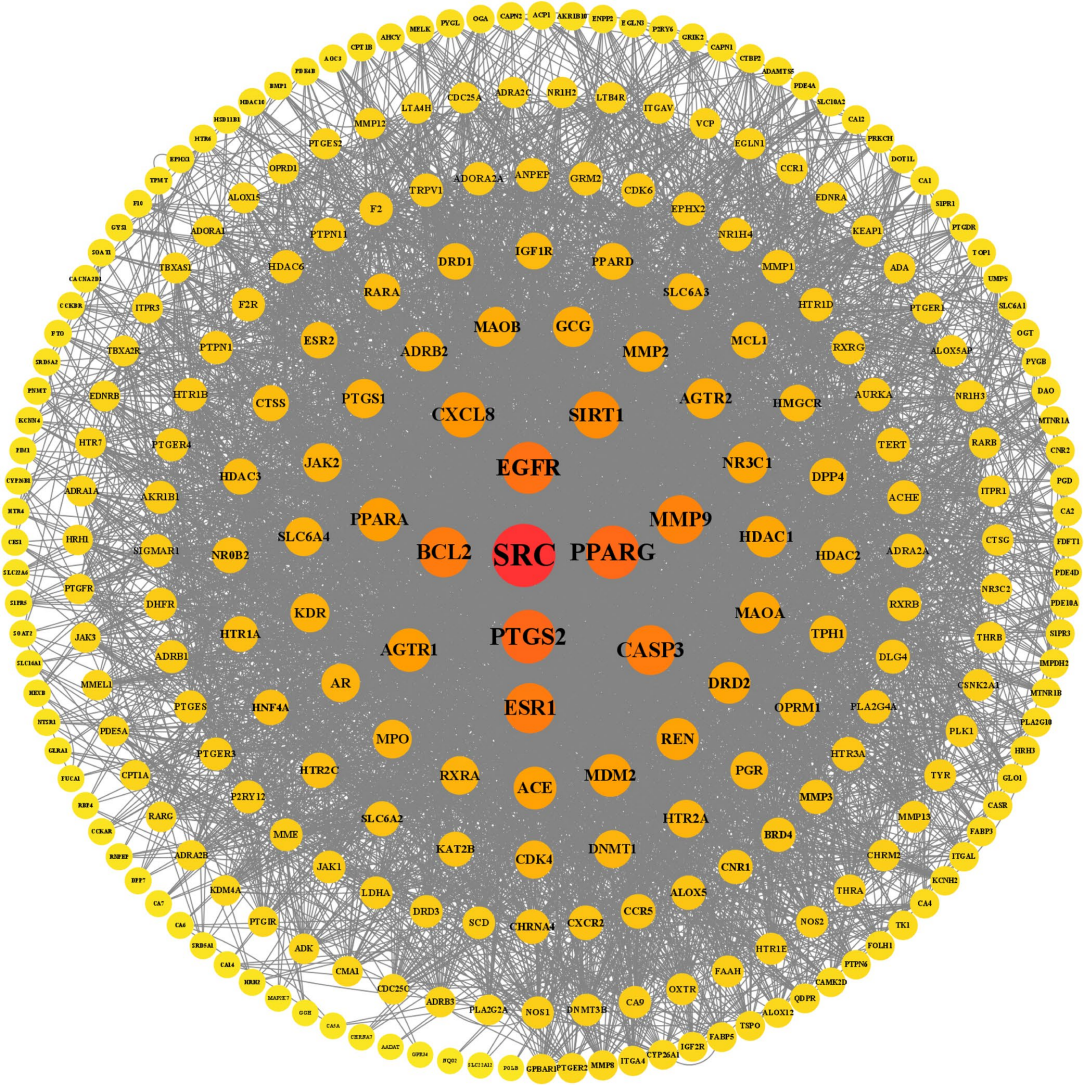
Protein–protein interaction network.

Finally, the SwissTargetPrediction database was used to search for the corresponding targets of differential metabolites in brain and intestinal tissues, and 326 targets were obtained. As shown in Figure 20, the main targets were SRC, PPARG, PTGS2, EGFR, ESR1, Bcl‐2, and Caspase 3. KEGG pathway analysis of the targets using the Metascape database yielded a total of 81 enriched KEGG signaling pathways, including neuroactive ligand‐receptor interactions, calcium signaling pathway, and PI3K/AKT signaling pathway. The results of this screening were consistent with ex vivo and in vivo studies on apoptosis‐related proteins, such as Bcl‐2, Caspase 3, and Bax, and the PI3K/AKT signaling pathway.

## 4 Discussion

The main cellular components of the hippocampal region are hippocampal neuronal cells, and their main roles include current memory, emotion regulation, and regulation of visceral functions. HT22 cells are a hippocampal neuronal cell line that have been widely used in a variety of studies, and their growth is sufficiently stable for in vitro models to study cognitive deficit mechanisms in AD. OA is a phosphodiesterase inhibitor that is commonly used to prepare neuronal cell injury models and was employed to induce HT22 cell damage, produce endotoxicity, and ultimately lead to neuronal cell apoptosis (Ravindran et al. 2011). Therefore, in this study, OA was used to induce HT22 injury, and DAP and DAP4 were identified as the active protein components that ameliorated OA‐induced HT22 cell injury by significantly increasing damaged cell viability. Hence, they were selected for subsequent experiments.

AlCl_3_/D‐gal co‐administration induces damage in AD mice; hence, it is a commonly used pharmacological animal model. Prolonged exposure to AlCl_3_ and D‐gal promotes the production of Aβ and *p*‐Tau, which induces a significant cognitive function decline, hippocampal organization abnormalities, oxidative damage, cholinergic dysfunction, and inflammatory responses in mice. This model mimics the biochemical and behavioral changes observed in AD; thus, it is a cost‐effective method for studying potential AD treatments and their mechanisms of action.

AD is characterized by cognitive dysfunction. In this study, we investigated the effects of cognitive dysfunction, that is, learning and memory, in AlCl_3_/D‐gal‐induced AD mice using an eight‐arm maze and MWM test. DAP and DAP4 administration significantly increased the escape latency of AD mice in the eight‐arm maze experiment. In the MWM experiment, DAP and DAP4 administration significantly shortened escape latency and increased the number of times AD mice traversed the platform position. Therefore, DAP and DAP4 administration improved the behavioral indices, learning memory ability, and exploration ability of AD mice.

Furthermore, cognitive dysfunction in AD is often accompanied by the formation of senile plaques resulting from excessive accumulation of Aβ and NFTs due to hyperphosphorylation of Tau protein. The abnormal accumulation of the 42‐residue long Aβ peptide (Aβ_1–42_) is considered the primary cause of the early onset of AD; hence, AD is considered as a protein misfolding disease characterized by a high propensity for aggregation (Goswamia et al. 2020). The enzyme immunoassay assay showed that DAP and DAP4 administration significantly reduced serum and brain tissue Aβ_1–42_ levels. Moreover, immunohistochemistry revealed Aβ_1–42_ expression in the CA1 and CA3 regions of brain tissue, which showed that Aβ_1–42_ expression was significantly increased in the AD model group and decreased after drug administration. BACE1 is an important enzyme that plays a key role in the formation of Aβ, and BACE1 promotes the catabolism of APP to produce Aβ. Western blot showed that DAP and DAP4 administration significantly downregulated APP, BACE1, and Aβ_1–42_ expression compared with that of the AlCl_3_/D‐gal model group. Moreover, DAP and DAP4 administration significantly reduced Aβ_1–42_ content and expression, which prevents APP catabolism and subsequent Aβ production by inhibiting BACE1 expression.

Tau is a protein associated with microtubules and plays a crucial role in maintaining the stability and organization of microtubule proteins within axonal and dendritic regions, thereby supporting the structural integrity of the neuronal cytoskeleton in the brain. However, aberrant hyperphosphorylation of Tau under pathological conditions disrupts its normal function, resulting in neuronal synaptic impairment and degeneration (Sinsky, Pichlerova, and Hanes 2021; Reddy 2011). When Tau undergoes hyperphosphorylation and exhibits diminished affinity for microtubule proteins, it accumulates in axons and neuronal cell bodies to form NFTs. The deposition of NFTs compromises physiological function, apoptosis, and neuronal degeneration, which advances disease progression and manifests as cognitive impairment and eventual cell death (Reddy and Reddy 2011; Rawat, Sehar, et al. 2022). Immunohistochemistry revealed significantly increased *p*‐Tau expression in the CA1 and CA3 regions of the brain tissue of AlCl_3_/D‐gal‐induced AD mice, which decreased after DAP and DAP4 administration. Western blot data demonstrated that DAP and DAP4 downregulated *p*‐Tau protein expression by decreasing the ratio of *p*‐GSK3β/GSK3β. Hence, DAP and DAP4 alleviated Tau hyperphosphorylation and improved intracellular NFTs by decreasing *p*‐GSK3β action.

Aβ accumulation and hyperphosphorylation of Tau protein had toxic effects on neurons, such as oxidative stress and activation of microglia, leading to various pathological events such as neuronal cell death in AD. Therefore, we examined oxidative stress, cholinergic damage, neuroinflammation, and apoptosis in AlCl_3_/D‐gal‐induced AD mice.

Oxidative stress causes neuronal damage via multiple pathways. Increased oxidative stress in the brain of patients with AD is closely associated with a variety of pathological manifestations, and the main causes include an imbalance of transition metal homeostasis in the brain, such as increases in the formation of Aβ‐bound species, activation and overexpression of related oxidative enzymes, as well as mitochondrial dysfunction in three areas (Ganguly et al. 2017, 2021; Dhapola et al. 2022). Oxidative stress arises from the dysregulation of pro‐oxidants and antioxidants within the body and can be classified into enzymatic and non‐enzymatic antioxidant categories. The brain utilizes these antioxidant mechanisms to protect against damage induced by reactive oxygen species (ROS). Under typical physiological conditions, ROS serve as crucial components of signaling pathways and transcriptional processes. However, the accumulation of ROS beyond the capacity of antioxidants leads to oxidative damage to cellular biomolecules and disruption of cellular homeostasis. This stimulates the production of pro‐apoptotic proteins by mitochondria, which ultimately induces neuronal apoptosis in the central nervous system (CNS) (Ferreira et al. 2015; Bai et al. 2022). In this study, AlCl_3_/D‐gal‐induced AD model mice showed a significant decrease in T‐SOD and GSH levels and a significant increase in MDA content, which were significantly reversed by DAP and DAP4 administration. Therefore, noxious stimuli can cause oxidative stress that can be alleviated by DAP and DAP4 administration.

The cholinergic system plays a significant role in numerous cognitive processes such as memory, learning, and emotional regulation. The development of AD symptoms is mainly linked to alterations in cholinergic synapses, elimination of certain types of ACh receptors, and the loss of ACh‐producing neurons, thereby resulting in cholinergic neurotransmission decline (Chen and Mobley 2019). ACh is a neurotransmitter in the cholinergic system and plays a regulatory role in the CNS. The neurotransmitter is produced from choline and acetyl coenzyme A in a single‐step process facilitated by choline acetyltransferase. AChE is a crucial enzyme in biological neurotransmission and is responsible for the breakdown of ACh in cholinergic synapses, which inhibits the excitatory effects of neurotransmitters on postsynaptic membranes. Therefore, inhibiting AChE activity is considered a potential therapeutic mechanism for alleviating AD symptoms (Sharma 2019). AChE and ACh levels in the serum and brain tissues of AlCl_3_/D‐gal‐induced AD mice were assessed using an enzyme immunoassay. These findings indicated a notable increase in AChE activity and a marked decline in ACh concentration in both the serum and brain tissues of the AD model group. In contrast, DAP and DAP4 administration increased ACh concentration and decreased AChE activity. This demonstrates that DAP and DAP4 support the cholinergic system, potentially exerting neuroprotective effects. In addition to their effects on the cholinergic system, DAP and DAP4 increase 5‐HT concentration in the serum and brain tissue and play a role in promoting neurotransmitter synthesis.

Neuroinflammation plays a crucial role in AD progression (Panda et al. 2024), with microglia and astrocytes in the CNS being the primary agents for investigating neuroinflammatory mechanisms (Daria et al. 2017; Singh et al. 2024). Microglia, which are natural immune cells in the brain, release inflammatory substances and remove waste and clumped proteins when they move to injured areas, causing damage to neurons, disruption in communication between nerve cells, and the buildup of harmful proteins (Leng and Edison 2021; Prinz, Jung, and Priller 2019). Astrocytes, a type of glial cells, are essential for supplying nutrients to neurons, creating neurotransmitters, promoting the formation of synapses and transmission of signals between neurons, and contributing to the blood–brain barrier (Yu, Nagai, and Khakh 2020). Moreover, microglia reactivate astrocytes and significantly contribute to neuroinflammation and neurodegeneration in AD. These findings highlight the collaborative functions of astrocytes and microglia. In this study, immunohistochemical experiments were conducted to examine Iba1 and GFAP levels in the CA1 and CA3 regions of AlCl_3_/D‐gal‐induced AD mice. A significant increase in the expression of both Iba1 and GFAP in the CA1 and CA3 regions of the AD model group was reversed by DAP and DAP4 administration. Administration of DAP and DAP4 may exert neuroprotective effects by suppressing inflammation.

The PI3K/AKT signaling pathway is widely involved in neuronal function, regulates a variety of cellular processes, such as survival, proliferation, and apoptosis, and has been implicated as an important upstream regulator of Nrf2 nuclear localization (Long et al. 2021; Ali et al. 2018). Nrf2 is a major regulator of the antioxidant response and plays a key role in the cellular antioxidant defense system due to its ability to regulate the expression of multiple antioxidant genes (Yang et al. 2021; Lee and Surh 2005). Under normal physiological conditions, Nrf2 is blocked in the cytoplasm and degraded by proteasome‐mediated processes; however, during stressful cellular events, Nrf2 enters the nucleus and binds to the antioxidant response element (ARE), triggering many protective pathways (Gugliandolo, Bramanti, and Mazzon 2020). Mechanistically, activation of the PI3K/AKT pathway allows Nrf2 to segregate from keap1, thereby promoting its nuclear translocation (Ali et al. 2018; Thangapandiyan et al. 2019). Nuclear Nrf2 binds to ARE and initiates the transcription of many antioxidant enzymes, including HO‐1 and NQO1, which are involved in the detoxification of free radicals, thereby maintaining cellular redox homeostasis (Vries et al. 2008; Hayes and Dinkova‐Kostova 2014). Evidence suggests that impaired Nrf2 function plays a role in the pathogenesis of cognitive dysfunction and AD. Branca et al. (2017) found that Nrf2 deficiency significantly exacerbates cognitive deficits, including spatial learning and memory, in an APP/PS1 mouse model. Moreover, Uruno et al. (2020) demonstrated that Nrf2 ameliorates cognitive deficits in an AD mouse model by inhibiting oxidative stress and neuroinflammation.

In this study, AlCl_3_/D‐gal induced a significant decrease in Nrf2 and HO‐1 expression in the CA1 and CA3 regions of the brain tissue of AD mice, and the administration of DAP and DAP4 upregulated the expression of Nrf2 and HO‐1 in the CA1 and CA3 regions. Furthermore, compared with the AlCl_3_/D‐gal model group, the western blot assay showed that DAP and DAP4 administration significantly upregulated Nrf2, HO‐1, NQO1, *p*‐PI3K, *p*‐AKT, and Bcl‐2 protein expression, while considerably downregulating keap1, Caspase 3, Caspase 9, Bax, and CytoC protein expression, and had no effect on PI3K and AKT protein expression. The results of this experiment were consistent with those of the in vitro experiments, indicating that DAP and DAP4 may play a role in improving AD by activating the PI3K/AKT/Nrf2 signaling pathway.

As shown by in vivo experimental studies, among the total protein and different protein fractions of sika deer antler, both DAP and DAP4 significantly improved AD. The improved effect of DAP4 was better than that of DAP, which may be related to the differences in structure and composition between DAP and DAP4. Amino acids are important precursors that form the primary structure of proteins and affect their structure and function. The chemistry of amino acid side chains has an important impact on protein folding and function, with hydrophobic amino acid residues being the most important drivers of protein folding and affecting biological activity. The amino acid content of different protein fractions was pre‐tested, and the hydrophobic amino acid content of the DAP4 was higher than that of DAP. Moreover, the lysine and alanine content were highest. Lysine promotes neurotransmitter synthesis, alanine maintains the normal function of the human nervous system, and glutamate and glycine are types of excitatory neurotransmitters. The levels of these four amino acids in DAP4 were significantly higher than those in DAP. A proteomic examination of total deer antler and different protein fractions was performed using liquid chromatography–tandem mass spectrometry. The analysis showed that four unique proteins, tyrosine protein kinase receptor, NADH–ubiquinone oxidoreductase chain 5, cytochrome b, and sphingosine‐1‐phosphate receptor 1, were present in DAP4 compared to the total DAP protein. Among them, tyrosine protein kinases have important roles in the nervous system, which can affect nervous system development by regulating cytoskeletal adequacy, regulating neuronal growth and differentiation, and are also involved in neural processes such as synaptic formation and transmission. NADH–ubiquinone oxidoreductase is a key component of the mitochondrial electron transport chain, and cytochrome b is also present in mitochondrial electron transport, which also corresponds to the results of this study, in which DAP4 played an ameliorating role in AD by inhibiting oxidative stress.

We further investigated the mechanism of action of DAP and DAP4 in improving AD through the microbe–gut–brain axis using 16S rRNA high‐throughput assays and metabolomics via ex vivo and in vivo investigations. Intestinal microbiology analysis showed that the intestinal flora of AD mice was disorganized, the species richness and diversity of the intestinal flora were significantly reduced, and the disorganization of the intestinal flora was ameliorated by DAP and DAP4 administration. The phyla were mainly dominated by Firmicutes, Bacteroidota, Verrucomicrobiota, and Proteobacteria (Backhed et al. 2005). Bacteroidota benefit the host by preventing infections by pathogens that colonize and infect the gut. In this study, Bacteroidota abundance was reduced in the intestinal flora of mice in the AD model group, and the administration of the drug reduced the relative abundance of Bacteroidota. Thus, DAP and DAP4 may ameliorate brain injury in AD mice by modulating Bacteroidota. This is consistent with previous studies, which reported that increasing the relative abundance of Bacteroidota may improve cognition. Zhao et al. (2023) found that nicotinamide mononucleotide played a role in protecting intestinal health and improving AD by increasing the production of short‐chain fatty acids and increasing the relative abundance of Bacteroidota. DAP and DAP4 increased the abundance of beneficial bacteria and decreased that of harmful bacteria. *Helicobacter* belongs to a specific classification of *Aspergillus* and is involved in the development of certain neurological disorders, with mechanisms of action involving neurotoxicity, neuroinflammation, and micronutrient deficiencies (Doulberis et al. 2020). This was also illustrated by the elevated *Helicobacter* levels in the intestinal flora of mice in the AD model group in this study. The administration of DAP and DAP4 restored *Helicobacter* abundance, suggesting that DAP and DAP4 played an ameliorative role in brain injury in AD mice by decreasing the abundance of the causative bacterium *Helicobacter*. The gut–brain axis is a key factor in controlling gut ecological dysregulation in neurological disorders (Pan et al. 2023); thus, further studies on gut and brain tissue metabolites have been conducted.

In this study, brain and intestinal tissue metabolites were analyzed using untargeted metabolomics. The analysis showed that there were 57 and 87 differential metabolites in DAP brain and intestinal tissues, respectively. There were 61 differential metabolites in DAP4 brain tissue and 106 differential metabolites in intestinal tissue. More metabolites were screened in the intestinal tissue than that in the brain, and the number of metabolites in DAP4 was greater than that in DAP.

After obtaining the metabolites in each tissue, the differential metabolites with trend changes were identified using STEM analysis. The main differential metabolites with trend changes obtained from brain tissues were: oleoyl‐2‐palmitoyl‐RAC glycerol, 1‐stearoyl‐lysine‐glycerol, 8‐hydroxyquinoline‐2‐carbonitrile, docosahexaenoic acid‐1‐stearoyl‐SN‐glycerol‐3‐phosphate ethanolamine, 2,4,6‐tri‐tert‐butylaniline, eicosatetraenoic acid, and thymosin β‐D‐glucoside negative ion. Differential metabolites with trend changes obtained from intestinal tissues were mainly: tetrahydrobenzyl alcohol, arachidonic acid‐2′‐fluoroethylamide, D‐red‐imidazolylglycerophosphate, 2‐amino‐2‐methyl‐1,3‐propanediol, 2‐bis[hydroxymethyl]‐2,2′,2′‐carbonitrile triethanol, DL‐preadrenaline, 2,4‐diaminotoluene, 3‐hydroxybutanoic acid, and L‐palmitoylcarnitine.

KEGG pathways were enriched for differential metabolites, and the main pathways enriched in the brain tissue were carbon metabolism, amino acid biosynthesis, pentose phosphate pathway, tyrosine metabolism, nucleotide sugar biosynthesis, cofactor biosynthesis, fructose and mannose metabolism, amino acid and nucleotide sugar metabolism, and purine metabolism. The main pathways enriched in the intestinal tissues were butyric acid metabolism, fatty acid degradation, steroid biosynthesis, tyrosine metabolism, primary bile acid biosynthesis, steroid hormone biosynthesis, glycine, serine, threonine metabolism, lipid metabolism, arginine and proline metabolism, unsaturated fatty acid biosynthesis, amino acid biosynthesis, arginine biosynthesis, aminocaproic acid tRNA biosynthesis, carbon metabolism, citric acid cycle (TCA cycle), pyruvate metabolism, and tyrosine metabolism.

The intestinal flora with changing trends obtained from the screening and the differential metabolites obtained from the screening were correlated and analyzed to obtain the intestinal flora–brain tissue metabolome pathway and the intestinal flora–intestinal tissue metabolome pathway, respectively. Ultimately, the signaling pathway co‐regulated by the brain and intestinal tissues was tyrosine metabolism. Tyrosine metabolism regulates abnormal energy metabolism, reduces inflammation, and regulates gut flora and neurotransmitters in AD therapy (Wang et al. 2021).

Screening of differential metabolite‐related targets showed that the main targets were SRC, PPARG, PTGS2, EGFR, ESR1, Bcl‐2, and Caspase 3. Furthermore, the KEGG pathway analysis mainly included neuroactive ligand‐receptor interactions, the calcium signaling pathway, and the PI3K/AKT signaling pathway. The results of this screening were consistent with in vivo and ex vivo studies on apoptosis‐related proteins, such as Bcl‐2, Caspase 3, and Bax, and the PI3K/AKT signaling pathway.

In this study, we used a combination of in vivo and ex vivo modeling to evaluate the efficacy of sika deer antler protein. We screened and obtained the active protein components and elucidated the mechanisms underlying the efficacy of deer antler protein in improving AD. The microbe–gut–brain axis and the analysis of intestinal microbiology and metabolomics revealed that deer antler protein played a role in regulating the abundance and diversity of intestinal flora. Moreover, correlation analysis of the intestinal flora–metabolomics pathway clarified that sika deer antler protein improved AD by regulating the tyrosine metabolism pathway and activating the PI3K/AKT/Nrf2 signaling pathway. Therefore, this study provides a novel mechanism by which sika deer antler protein improves AD.

## Author Contributions

**Lei Li:** data curation (equal), investigation (equal), methodology (equal), writing – original draft (equal). **Lulu Wang:** investigation (equal), methodology (equal). **Weixing Ding:** resources (equal). **Jianfa Wu:** methodology (equal). **Fei Liu:** investigation (equal). **Jiansong Liu:** methodology (equal). **Jing Zhang:** funding acquisition (equal), methodology (equal), supervision (equal), validation (equal), writing – review and editing (equal). **Jing Wang:** project administration (equal).

## Ethics Statement

This study was approved by the Institutional Review Board of Jilin Agricultural University Laboratory Animal Welfare and Ethics Committee.

## Consent

Written informed consent was obtained from all study participants.

## Conflicts of Interest

The authors declare no conflicts of interest.

## Data Availability

The authors have nothing to report.
